# Acknowledgement to Reviewers of the *International Journal of Molecular Sciences* in 2017

**DOI:** 10.3390/ijms19010303

**Published:** 2018-01-19

**Authors:** International Journal of Molecular Sciences Editorial Office

**Affiliations:** MDPI AG, St. Alban-Anlage 66, 4052 Basel, Switzerland

Peer review is an essential part in the publication process, ensuring that the *International Journal of Molecular Sciences* maintains high quality standards for its published papers. In 2017, a total of 2778 papers were published in the journal. Thanks to the cooperation of our reviewers, the median time to first decision was 18 days and the median time to publication was 43 days. The editors would like to express their sincere gratitude to the following reviewers for their time and dedication in 2017:

Abbott, Karen L.Lopes, SusanaAbdalla, Maher Y.Lopez De Diego, MartaAbd-El-Aziz, AlaaLópez, AliciaAbenavoli, LudovicoLopez, José Manuel BlancoAbend, MichaelLópez-Andrés, NataliaAbizaid, AlfonsoLopez-Camarillo, CesarAbou Neel, Ensanya A.Lopez-Escamez, Jose A.Abraham, DietmarLópez-Moya, Juan JoséAbuelo, ÁngelLophatananon, ArtitayaAburima, AhmedLora, JairoAbu-Romman, SaeidLord, JanetAccornero, FedericaLorenzini, AntonelloAchinger-Kawecka, JoannaLorenzo, ÓscarAchour, BrahimLorey, FredAcuña-castroviejo, DarioLorico, AurelioAdachi, HiroakiLorson, ChristianAdam, VirgileLortat-Jacob, HuguesAdam, VojtechLos, MarekAdams, JamesLossius, AndreasAdams, Michael A.Lötsch, JörnAdan, RogerLou, SongAddis, Maria FilippaLou, YonggenAdhikari, NeetaLoutradis, DimitriosAdunyah, Samuel E.Louveau, IsabelleAeby, EricLove, JohnAfosah, DanielLowe, MartinAfrin, SadiaLowe, RohanAgarwal, GauravLoxham, MatthewAgarwal, ShivangiLozano, Jose ManuelAgeitos, JoseLu, JunAggarwal, AbhishekLu, LinchaoAggarwal, Bharat B.Lu, ShuoAgostoni, CarloLu, Wan-JungAgudelo-Romero, PatriciaLu, YangAguilo, FrancescaLu, ZhanpingAh Kim, HyunLuca, MariaAhmad, ZeeshanLucacchini, AntonioAhmadi-Afzadi, MasoudLucarini, GuendalinaAhmed, HafizLuce-Fedrow, AlisonAhmed, MaryaLucena, María IsabelAhn, Ji-YoungLucena, RafaelAhnert, PeterLuchetti, MicheleAhrens-Nicklas, Rebecca C.Luciano, Alberto MariaAimanianda, Vishu KumarLuckenbach, TillAiras, LauraLuco, Reini F.Aires, AlfredoLuconi, MichaelaAiroldi, CristinaLüder, Carsten G. K.Aithal, GuruLudueña, RichardAitor, Nogalez-GonzalezLudwiczuk, AgnieszkaAkamatsu, MikiLuft, ThomasAkhtari, MassoudLuís, ÂngeloAkiba, EtsukoLukas, JanAkimov, Sergey A.Lukashevich, Igor S.Akita, SadanoriLukaszewski, AdamAl Massadi, OmarLundstrom, KennethAla, UgoLuo, HuachengAl-Aubaidy, H.Luo, LynaAlavez, SilvestreLuongo, LivioAlbano, EmanueleLupien, AndréanneAlbi, ElisabettaLuque, FranciscoAlbrecht, Eric A.Łuszczek-Trojnar, EwaAlbrecht, RandyLutfy, KabirullahAlcaide, PilarLuthra, AmitAldamiz-Echevarria, LuisLuu, Doan-trungAl-Dujaili, EmadLy, HinhAlemany, SilviaLymperopoulos, AnastasiosAlessandro, RiccardoLynce, FilipaAlexander, DenisLyng, Maria BAlexandre, MezentsevLyons, LisaAlexeyev, MikhailLyons, Paul E.Alexeyev, Mikhail F.Lystad, Alf HåkonAlexiou, ChristophM. Peffley, DennisAlfano, DanielaM.Fuller, PatrickAlfano, MassimoM.L. Hutnik, CindyAl-Hendy, AymanMa, GuojiaAlique, MatildeMa, QingAlivernini, StefanoMa, Wen-JuanAllardyce, ClaireMa, XiaochaoAllegra, SarahMa, XingzheAllen, ChristopherMa, YonggangAllen, Margaret L.Ma, YuAllione, AlessandraMa, ZexuAllnutt, TheoMaak, SteffenAlmasan, AlexMacabeo, Allan P.Al-Mashhadi, Rozh H.Macchi, BeatriceAlmeida, PedroMacDonald, Clinton C.Almouzni, GenevieveMacdonald, Stephen P. J.Alonso, SantosMacedo, RodrigoAlonso-Magdalena, PalomaMachado, Mariana V.Alshbool, Fatima Z.Machireddy, NarsaAltaner, CestmirMacia, AngelaAlteri, Christopher J.Macias, Maria J.Altieri, BarbaraMaciejewska, DominikaAltomare, ClaudiaMack, Andreas F.Altomonte, JenniferMacvittie, Thomas J.Alvarez, CatalinaMadabhushi, RamAlves, CelsoMadan, JulietteAlves, SandraMadeleine, Margaret M.Alwahsh, SalamahMadhukar, BurraAlwood, Joshua S.Maeda, HiroshiAmaral, AlexandraMaekawa, FumihikoAmarowicz, RyszardMaekawa, ShinyaAmasheh, SalahMaestro, Miguel A.Amati, FrancescaMagnaghi, ValerioAmbati, Ranga RaoMagnaldo, ThierryAmbigapathy, GaneshMagni, FulvioAmeri, P.Magnusson, Karl-EricAmicarelli, FernandaMahajan, SahilAmini-Nik, SaeidMahajan, Vinit B.Amirkhani, MasoumeMahajna, JamalAmit, TiwariMahimainathan, LeninAmital, HowardMahmood, JavedAmorim, Maria JoãoMahoney, Sara E.Amorós, AsunciónMahony, Timothy JohnAmpatzis, KonstantinosMahsoub, HassanAmpuero, JavierMaier, PatrickAmreddy, NarsireddyMaillard, VirginieAn, SaiMaioli, MargheritaAnanthakrishnan, Soundaram JeevarathinamMaione, FrancescoAnastasi, EmanuelaMai-Prochnow, AnneAnastasiadou, MariaMaiti, AparnaAnczukow-Camarda, OlgaMajumdar, IndrajitAndersen, Thomas LevinMajumdar, SudiptaAnderson, Travis M.Majumder, MousumiAndersson, EmmaMakarevitch, IrinaAndjelkovic, Anuska V.Makena, MonishAndo, HitoshiMakishima, MakotoAndolfo, AnnapaolaMalemud, Charles J.André, VâniaMalentacchi, FrancescaAndreassi, Maria GraziaMalhotra, VivekAndreasson, ErikMalkoc, VeysiAndreoni, FrancescaMalli, RolandAndreozzi, Giuseppe MariaMallipeddi, Prema LathaAndres, Allen M.Mallo, FedericoAndrés, Maria FeMalloci, GiulianoAndrews, ZaneMalmström, LarsAndrey, GuillaumeMaloyan, AlinaAndrieux, AnnieMalpeli, GiorgioAndrukhov, OlehMamedov, TarlanAnes, ElsaMammoto, TadanoriAngelo, AlbertiMan, DulaAngelova, AngelinaMancheño, JoséAngelovich, TomMancias, Joseph D.Anglister, JacobMandapati, R. R.Aniello, FrancescoMandava, NandaAnifandis, GeorgeMandolesi, GeorgiaAnjos, OféliaMandrioli, MauroAnkiewicz, AdrianMangiatordi, Giuseppe FeliceAnnadurai, AnandhanMann, FrancisAnower, RokebulMann, Karen P.Ansquer, Jean ClaudeMannell, HannaAntal, Diana SimonaManni, AndreaAntaris, Alexander LeeManninger, MartinAnthony, KarenManno, CarloAntognelli, CinziaManocha, GunjanAntoine, Marie-HélèneManor, DannyAnton, SteveMansky, Kim C.Antonelou, Marianna H.Mantzioris, EvangelineAntoniadi, IoannaManzardo, A.M.Antoniak, SilvioManzella, LiviaAnzenbacher, PavelMao, MengAon, Miguel A.Marasco, DanielaAoyama, KojiMarc, DanielAparicio, FredericMarcal, HelderApone, FabioMarchant, DavidApplegate, Lee AnnMarchese, AdrianoApte, UdayanMarchetti, MartaArdley, JulieMarchini, SergioArduini, AlessandroMarci, RobertoArena, FrancescaMarcinkowska, EwaArenas, JesúsMarco, Eva MaríaArens, PaulMarcovici, GenoArent, CamilaMardente, StefaniaArese, MarziaMaréchal, AlexandreArgia, KatsuhikoMaréchal-Drouard, LaurenceArianna, MaruccoMárialigeti, KárolyAriga, HiroyoshiMarikawa, YusukeAriyasu, DaisukeMarin, Jose J. G.Ariza, Maria EugeniaMarincs, FerencArnbjörnsson, EinarMariniello, LoredanaArndt, Patrick G.Marino, AngelaArnould, ThierryMarino, JosephAronowitz, Joel A.Mar­itschnegg, Elis­a­bethAronson, MelyssaMarkiv, AnatoliyArrabal, PilarMarkkanen, EnniArteaga, OlatzMarklund, UlrikaArtioli, GraziaMarkov, Marko S.Artlip, TimothyMarlow, Florence L.Arulselvan, PalasamyMarnetto, FabianaArumugam, SomasundaramMarrs, James A.Asa, SylviaMarsich, EleonoraAsahara, HiroshiMarsolais, FrédéricAsahi, MichioMartí, SergioAsamizu, ShumpeiMartin, AbrahamAsano, RyutaroMartin, CathieAsano, TomoichiroMartin, FinianAsatryan, RubikMartin, FranciscoAsensi, VictorMartin, JessicaAshiuchi, MakotoMartin, Luc J.Askarian-Amiri, Marjan E.Martin, Thomas JohnAslam, MuhammadMartina, KorfeiAslanidis, CharalamposMartín-Acebes, Miguel A.Assanelli, DeodatoMartín-Antonio, BeatrizAssenat, EricMartín-Aragón, SagrarioAstolfi, StefaniaMartineau, YvanAsuero, Agustín G.Martinelli, PaolaAtes-Alagoz, ZeynepMartínez De Paz, Pedro JoséAthyros, VasiliosMartínez, AntonioAttanasio, ChiaraMartinez, ManuelAttar, HooyarMartinez, VicenteAtteritano, MarcoMartínez-Campa, CarlosAttila, AdamMartínez-Esteso, María JoséAudette, Gerald F.Martínez-Gómez, PedroAudrey, VarinMartinez-Lage, AndresAugé, NathalieMartin-Fontecha, AlfonsoAuguet, TeresaMartín-Hernández, DavidAugusto, OrlandiMartini, ClaudiaAustin, RachelMartini, MaurizioAuvynet, ConstanceMartínková, LudmilaAvato, PinarosaMartino, SabataAvci-Adali, MeltemMartinoli, Maria-GraziaAverbeck, DietrichMartins, AlbinoAviles, Francesc XavierMartins, Ian JamesAvni, AdiMartins, NatáliaAvrova, Natalia F.Martorana, AlessandroAy, FerhatMaruf, Abdullah AlAydemir, TolunayMarullo, StefanoAyers, DuncanMaruyama, KeiAyuso, Maria IreneMarverti, GaetanoAzarnia Tehran, DomenicoMarvin, ShaunaAzevedo, HerlanderMarycz, KrzysztofAzhar, MohamadMarzban, HassanAzimzadeh, OmidMarzocco, StefaniaAzuma, KagakuMarzullo, PaoloAzuma, KazuoMasaki, TsutomuBaba, HideoMaserti, Bianca ElenaBaba, YuhMaserti, BiancaelenaBabu, AnishMashima, RyuichiBach, AndreasMasiello, PellegrinoBache, SørenMasieri, FedericaBack, K.Masli, SharmilaBacker, Marina V.Masłyk, MaciejBackman, LudvigMasquida, B.Baczek-Kwinta, RenataMassot, MalenBado, IgorMasters, Colin L.Bae, Hae-RahnMastrangelo, Anna M.Baek, Kwang-HyunMastrocola, RaffaellaBaenas, NievesMastropasqua, LeonardoBaer, Patrick C.Masuda, KiyoshiBaeumer, WolfgangMasuda, ShinjiBagchi, RammyaniMasui, KentaBahar, EntazMasureel, MatthieuBai, HuaMateos-Timoneda, M. A.Bai, Li-YuanMateu, EnricBaier, MargareteMathesius, UlrikeBairaktari, Eleni T.Matheu, AnderBajpai, RichaMatic, MajaBakay, MarinaMatos, Augusto J. F.Bakovic, MaricaMatos, ManuelaBaksh, ShairazMatsubara, KeiichiBakunina, IrinaMATSUGAKI, AiraBal, WojciechMatsui, MasakiBalato, AnnaMatsumoto, Ken-ichiroBaldini, EnkeMatsumoto, KunioBaldwin, CynthiaMatsumoto, YasuhikoBalestrini, Raffaella MariaMatsuo, MuneakiBall, JonathanMatsushita, TakehikoBallerini, ClaraMatsuura, BunzoBallizany, WouterMatta, CsabaBally, JuliaMatta, JaimeBally, MartaMattei, BenedettaBamdad, CynthiaMattei, FabrizioBamford, ConnorMatteoli, GianlucaBandini, ErikaMattner, JochenBanerjee, Hirendra NathMatwijczuk, ArkadiuszBanerjee, KalpitaMaura, DamienBanerjee, ParthaMauriello, FrancescoBanfalvi, ZsofiaMaury, EleonoreBang, Marie-LouiseMautner, AndreasBaniahmad, AriaMavridis, KonstantinosBanilas, GeorgiosMavrogonatou, EleniBannard-Smith, JonathanMawhinney, ThomasBanning, AntjeMaxel, TrineBansal, RuchiMaximiano, Marisa Da SilvaBanti, Christina N.Maxwell, AnthonyBao, YongpingMaxwell, Jessie R.Baptiste-Roberts, KeshaMay, FelicityBarabas, PeterMayer, Gregory D.Barabutis, NektariosMayhan, William G.Baragetti, AndreaMayne, ChristopherBarata, JoãoMazur-Bialy, Agnieszka IrenaBarbagallo, DavideMcArdle, StephanieBarbe, Anna GretaMcCallum, Jason L.Barbe, Mary F.McCormick, CraigBarber, MelissaMcCoy, Sarah J. BreeseBarbieri, AntonioMcDaid, HayleyBarbu, Sorin T.McDaneld, Tara G.Barcelos, GustavoMcDermott, LindsayBárcena, José AntonioMcDermott-Roe, ChrisBarchetta, IlariaMcDonagh, BrianBärebring, LinneaMcdonald, CourtneyBarilli, AmeliaMcDonald, KarenBarisani, DonatellaMcFeeters, Robert L.Barrajón-Catalán, EnriqueMcGowan, EileenBarrea, LuigiMcGrail, MauraBarros, PedroMcintosh, RobertBarry, CarolMcIntyre, Jenifer K.Barta, CsengeleMcKay, BrianBartelt, AlexanderMcKeague, MaureenBartholomeusz, GeoffreyMcKenna, Malachi J.Bartley, JamesMcKim, Kim S.Bartocci, PietroMcLean, Robert J. C.Bartolazzi, ArmandoMcLemore, Gabrielle LynnBartolini, ManuelaMcloughlin, P.Bartosz, GrzegorzMcMahon, FrancisBartoszewski, RafalMcMahon, Hilary E.M.Barzilay, Joshua I.McMenamin, MarkBar-Zvi, DudyMcMillin, MatthewBasak, DebasishMcmorrow, TaraBasar, MuratMcMurray, FionaBasavarajappa, Balapal S.Meacci, ElisabettaBascom, GavinMeade, KieranBasiak, EwelinaMeadows, ShannonBasini, GiuseppinaMediati, Rocco DomenicoBasolo, FulvioMedina Piles, VicenteBasso, KariMehta, Akul Y.Bastarrachea, Luis J.Mehta, PoojaBasu, AbhijitMehta, ShwetalBasu, AlakanandaMehta, StutiBasu, SujitMei, Ya-FangBatorova, AngelikaMeierhofer, DavidBatsché, EricMeijer, H. J. G.Battino, MaurizioMeijles, Daniel N.Battistelli, CeciliaMeinke, PeterBattistelli, MichelaMelchor, JuanBattisti, JamesMeldolesi, JacopoBaucher, MarieMeli, AlbanoBaud, OlivierMelis, AnastasiosBauer, GeorgMelkani, GirishBauer, JohannMellor, Andrew L.Baulch, Janet E.Melnik, BodoBawadekar, MandarMelo, SoniaBayat, VafaMeloni, GabrieleBayin, N. SumruMelville, StephenBazafkan, HodaMenand, BenoîtBazhanova, Elena D.Menassa, RimaBazihizina, NadiaMendonca, AntonioBažok, RenataMendonça, NunoBeatriz, Abós GraciaMenet, Marie ClaudeBeatson, Richard E.Meng, DongBeberok, ArturMeng, WeihuaBeck, AndreasMenkhorst, EllenBeckenbach, AndrewMenon, VipinBecker, ChristophMenu, ElineBecker, KathrinMercati, FrancescoBecker, Therese M.Merry, TroyBecuwe, PhilippeMeruvu, SunithaBedia, CarmenMeschwitz, SusanBedogni, FrancescoMessa, PiergiorgioBeer, RossMessaritakis, IppokratisBehera, SmrutisanjitaMessini, Christina I.Beilby, MaryMessinis, IoannisBeisswenger, ChristophMessner, BarbaraBeitz, EricMetharom, PatBejarano, IgnacioMettananda, SachithBekinschtein, PedroMetzinger, LaurentBekri, SoumeyaMeulia, TeaBelden, William J.Meurens, FrançoisBelgacem, Yesser H.Mexas, Angela MarieBelide, SrinivasMeyer, GregoryBell, AndrewMézes, MiklósBellés, José MaríaMező, GáborBellosta, PaolaMiao, JiBelluzzi, AndreaMiao, YinglongBelov, GeorgeMiccadei, StefaniaBełtowski, JerzyMichael, GroszmannBelyaeva, NataliaMichaeloudes, CharalambosBen Mahmoud, LobnaMichael-Titus, Adina TeodoraBenaiges, Maria D.Michalek, KatarzynaBen-Dov, Iddo Z.Michalska, AnnaBenedetti, MicheleMicheau, OlivierBenigni, ArielaMicheletti, GabrieleBenjamin, DonMichelhaugh, Sharon KayBenner, ChrisMichetti, FabrizioBenno, WeigmannMichiels, CarineBenoist, HervéMichigami, ToshimiBenowitz, Larry I.Mico, Juan AntonioBentel, Jacqueline M.Midgley, AdamBenzeroual, Kenza E.Midgley, Adam C.Berbari, NicolasMieda, MichihiroBerdis, Anthony J.Miernyk, Jan ABerecki, GezaMietlicki-Baase, Elizabeth G.Berenbrink, MichaelMiguel, Leiva-BrondoBerent-Maoz, BeataMíguez, Jesús M.Bergen, WernerMihailiasa, ManuelaBerger, Ralf G.Mi-ichi, FumikaBergeron, E.Mikel, ZaratieguiBergheim, InaMikhailova, AlexandraBergquist, RobertMiki, KojiBermudez, MarcelMiki, YasuhiroBernardi, FrancescoMiklossy, GabriellaBernasconi, PiaMikula, MarioBernitsas, EvanthiaMilanesio, MarcoBernstein, Hans-GertMilano, FrancescoBerruyer, RomainMilej, DanielBerski, WiktorMilhinhos, AnaBerthelot, KarineMilic, NatasaBerthelot, LaurelineMilione, MassimoBerthod, FrançoisMillar, J. CameronBertinaria, MassimoMiller, Aaron W.Bertok, TomasMiller, JohnBertoli, CosettaMiller, Judith S.Bertoli, GloriaMillet, OscarBertolini, MartaMills, StuartBertoni, GiuseppeMilner, JoelBesnard, GuillaumeMimaki, YoshihiroBevan, DavidMin, BaehyunBeverly, Levi J.Min, Jung-joonBeversdorf, DavidMinami, YoichiBeyer, Andreas M.Minchin, JamesBhadauria, VijaiMine, YuichiBhattacharyya, GargiMinervini, GiovanniBhaumik, AnusarkaMing-Wei, LinBhuiyan, Md. ShenuarinMino, MasanobuBhutani, NidhiMinocha, Subhash C.Bhutta, MahmoodMinor, PhilipBi, XinMinutolo, FilippoBiagi, MarcoMiotto, BenoitBiagini, GiuseppeMiousse, IsabelleBian, LiMiquel, M.Biasi, FiorellaMiragoli, MicheleBichet, Daniel G.Mirali, MahlaBichko, VadimMiranda, CristobalBicker, GerdMiranda-Castro, RebecaBickerton, EricaMireau, HakimBid, HemantMirey, GladysBiddle, AdrianMiri, Amir K.Bideshi, DennisMiron, Richard J.Bidwai, AshokMiroshnichenko, AndreyBiedermann, ThomasMirzayans, RazmikBielak-Zmijewska, AnnaMishin, Alexander S.Bienkiewicz, Ewa AnnaMishra, JayshreeBiernasiuk, AnnaMishra, NigamBiersack, BernhardMiteva, MariyaBijak, MichałMithöfer, AxelBijlard, EvelineMitoma, HirokiBijlsma, Maarten F.Mitra, AbhisekBikiaris, DimitriosMitrofanis, JohnBilbao, Jose RamonMitroulis, IoannisBilger, AndreaMitsuya, ShiroBingol, KeremMiura, KazukiBinstadt, BryceMiura, SayakaBiondani, GiuliaMiyaji, HirofumiBirch, JohnMiyake, MakitoBird, AmandaMiyamoto, KojiBird, RanjanaMiyamoto, ShingoBirkemeyer, ClaudiaMiyanoiri, YoheiBirrell, MarkMiyata, AtsuroBisaga, MaciejMiyata, YoshihikoBisaglia, MarcoMiyoshi, HiroakiBischof, JoachimMiyoshi, NorioBishehsari, FarazMizobata, TomohiroBishop, DouglasMizuno, Cassia S.Bisignano, GiuseppeMizuno, MasashiBišová, KateřinaMlejnek, PetrBisson, JonathanMlera, LuwanikaBiswas, SantanuMo, Jung-SoonBitler, BenjaminMoal, IainBittner, StefanMoar, William J.Bizzarri, MarianoMobed-Miremadi, MaryamBjörnsson, Einar StefánMocan, AndreiBlack, Samuel J.Mocellin, SimoneBlais, AnneModahl, CassandraBlanco-Aparicio, CarmenModhiran, NaphakBlanco-Ulate, BarbaraModos, DezsöBlando, FedericaMoeller, Hanne B.Blank, VolkerMoertl, SimoneBlankesteijn, W. MatthijsMogi, MasakiBlasiak, JanuszMohamed, IslamBlatt, MichaelMohamedali, KhalidBlomme, JonasMohammad, Haroon TajBlum, RobertMohammed-Geba, KhaledBlumenthal, EdwardMohanta, TapanBlumer-Schuettea, Sara E.Möhlhenrich, S. C.Boareto Do Amaral, MarceloMohr, StefanBobek, VladimirMok, Daniel Kam-WahBocchio-Chiavetto, LuisellaMoldawer, Lyle L.Boccuto, LuigiMolfino, AlessioBochman, Matthew L.Mollapour, MehdiBoczek, TomaszMollen, Kevin P.Bode, Robert F.Møller, Ian MaxBodnar, LubomirMolnar, FerdinandBoege, FritzMolugu, Sudheer KumarBoekema, BoukeMolyneux, KarenBoerma, MarjanMoncayo, RoyBogado Pascottini, OsvaldoMonchau, FrancineBogdanowicz, KrzysztofMongiat, MaurizioBogdański, PawełMonk, JenniferBoggaram, VijayMonroy, FernandoBogliolo, MassimoMontani, Jean-PierreBogni, AlessiaMonteiro, SusanaBøgwald, JarlMonteith, G. R.Bohlson, Suzanne S.Montenegro, LuciaBohutskyi, PavloMontesarchio, DanielaBoisvert, François-MichelMonteys, Alex MasBojková, BiankaMontoya, GuillermoBolger, AnthonyMoon, Byeong CheolBolibok-Brągoszewska, H.Moon, IlsooBolino, AlessandraMoore, Axel C.Bollag, Wendy B.Moore, Thomas FBolle, CordeliaMoos, PhilipBölter, BettinaMorales, JulioBombieri, CristinaMorales, PaulaBonen, LindaMorales-Cruz, AbrahamBongers, KaleMorales-González, J. A.Boogaard, PeterMorales-Tirado, VanessaBoomer, Jonathan S.Morandi, LucaBopegamage, ShubhadaMorbidelli, LuciaBorges, João P.M.R.Moreira, IrinaBorges, KarinMoreira-Teixeira, LúciaBorghaei, Ruth C.Moreno, Carlos S.Borghini, AndreaMoreno, MargaritaBorkow, GadiMoreno, MariaBorkowski, LeszekMorgat, ClémentBorodinsky, Laura N.Mori, KazuhiroBorovecki, AnaMoriarty, Thomas FintanBorrego, FranciscoMoriguchi, TakayaBorroni, RiccardoMorikawa, SatoruBorst, PietMorikawa, ToshioBorucki, MonicaMorimoto, TatsuyaBoryczka, StanisławMorino, KatsutaroBoscaiu, MonicaMorioka, TomoakiBoschetti, FedericaMorita, HiroyukiBose, DebojitMorita, ManabuBose, SayantanMorley, Alexander A.Bosman, Giel J. C. G. M.Morley, Steven DouglasBosnjak, BerislavMoro, LoredanaBosse, Jens B.Moro, StefanoBossi, FleurMorotti, AlessandroBotelho, Monica C.Morreale, MarcoBotha, FrederikMorris, Andrew J.Boto, LuisMorris, Claudia R.Bottari, FabioMorris, James L.Bottaro, Donald P.Morrison, DouglasBottier, CélineMorrison, MelanieBouattour, MohamedMorris-Rosendahl, DeborahBouckaert, JulieMorrissey, JeremiahBourdon, EmmanuelMorroni, FabianaBouropoulos, NikolaosMorrow, JenniferBousbaa, HassanMortiboys, HeatherBoutin, JeanMortier, JérémieBoutin, Jean AlbertMorton, DavidBouttier, ManuellaMortusewicz, OliverBouvier, BenjaminMoschetta, MicheleBowen, JamesMoschovi, MariaBowen, RichardMoser, Lindsey A.Bowerman, MelissaMossialos, DimitrisBowling, HeatherMotta, AntonellaBowsher, Alan W.Mottes, MonicaBoyd, ChrisMourad, RaphaelBoyer-Guittaut, MichaëlMourtas, SpyrosBoysen, PrebenMousa, Shaaban A.Bozic, JoskoMoustaid-Moussa, NaimaBozzaro, SalvatoreMovahedi, AliBozzi, AdrianaMoxon, JosephBozzi, Fabio LuigiMuckle, Catherine AnneBrábek, JanMueller, GeoffreyBracci, MassimoMueller, Wolf C.Bradbury, AllisonMuenzer, JosephBramanti, VincenzoMufson, Elliott J.Branchini, AlessioMuggia, FrancoBrandão, Rita DiasMuinelo-Romay, LauraBrandhorst, BruceMukaida, NaofumiBratlie, Kaitlin M.Mukherjee, SomnathBravo López, Susana B.Mulas, MaurizioBreitenbach Barroso Coelho, Luana CassandraMulatero, PaoloBreitenfeld Granadeiro, LuizaMulinacci, NadiaBrender, JeffreyMüller, MarkusBrennan, StephenMuller, Patricia A. J.Brenner, ThorstenMüller, TimoBrestic, MarianMulo, PaulaBreves, GerhardMunang’andu, H. M.Brewer, MeganMunday, MichaelBricknell, IanMuñoz-Barroso, IsabelBridges, ChristyMunoz-Fontela, CesarBridgewater, DarrenMunro, CarolBrier, Michael E.Mura, UmbertoBrierley, IanMurai, JunkoBrinchmann, Monica F.Murakami, SeishiBrini, MarisaMurakami, YasufumiBrochot, AmandineMurakami, YoshikiBroderick, Tom L.Murase, SachikoBröer, StefanMurata, TakayukiBronisz, AgnieszkaMurata, YoshiyukiBrooks, JamesMurayama, TakashiBross, PeterMuresan, VladBroszczak, DanielMurono, ShigeyukiBroussard, GregoryMurotomi, KazutoshiBrown, Euan R.Murphy, AndrewBrown, JamesMurphy, PaulaBrown, Keith W.Murray, Deirdre M.Brown, Robert J.Murray, RachaelBrown, StephenMurray, Thomas ScotBrowning, Glenn F.Murthi, PadmaBrugnera, EnricoMuruve, DanielBrullo, ChiaraMusch, MarkBrumeanu, Teodor-DoruMustafa, GhazalaBrummelte, SusanneMutti, LucianoBrumos Fuentes, JavierMyers, MarkBrunasso, A. M. G.Myers, StephenBrunner, AndreasMyllykallio, HannuBruns, HeikoMyśliwiec, HannaBruschi, FabrizioMysliwiec, TamiBrusslan, Judy AMystkowska, JoannaBrzozowski, TomaszNagahashi, MasayukiBubb, KristenNagai, KatshitoBubici, GiovanniNagai, KatsuraBublitz, MargaretNagai, KouheiBubulya, Paula A.Nagai, NoriakiBucciantini, MonicaNagai, ShigenoriBuchet, ReneNagai, YoshinoriBuchheim, MarkNagaraj, ChandranBucki, RobertNagaraja, Mamta PatelBuckley, Bradley A.Nagarajan, SureshbabuBuckley, M.Nagaraju, G. P.Bucolo, ClaudioNagata, EiichiroBudak, HikmetNagoya, SatoshiBuddaseth, SalmaNagwekar, JanhaviBudka, HerbertNagy, EndreBuechler, ChristaNagy, IstvanBuellesbach, JanNagy, LaszloBueno, MartaNahashon, SamuelBuettner, AndreasNair, VimalBugge, ThomasNajimi, MustaphaBugla-Płoskońska, GabrielaNakagawa, TakuroBugliani, MarcoNakagawa, YoshimiBullon, PedroNakahama, Ken-ichiBultynck, GeertNakajima, HirokoBumgardner, Joel D.Nakaki, ToshioBum-Soo, HahnNakamoto, ShingoBunker, AlexNakamura, MasatoBuonerba, CarloNakamura, NobuhiroBuono, P.Nakamura, Shun-ichiBuonocore, DanielaNakamura, TsukasaBurdo, Tricia H.Nakanishi, AkiraBurger, MichaelNakano, ToshiakiBurgess, John A.Nakashiro, Koh-IchiBurghardt, NeshaNakayama, YujiBurghardt, RobertNakchbandi, InaamBurgstaller, JoergNalivaeva, NataliaBurke, MarkNallasamy, PalanisamyBurow, MeikeNalvarte, IvanBurrin, Douglas G.Nam, ONG ChoonBurt, TalNamgaladze, DmitryBurtea, CarmenNanavati, CharviBurtey, StéphaneNanba, DaisukeBus, James S.Nandakumar, Kutty SelvaBusardo, FrancescoNandha Premnath, P.Buscarinu, Maria ChiaraNandi, SaikatBusciglio, JorgeNandwana, VikasButler, MarkNanjappa, ManjunathaButlin, MarkNanni, LorisButtenschoen, KlausNapierala, MarekButterfield, Russell J.Napoletano, ChiaraBüttner, HartwigNapoli, Pietro EmanueleByun, JonghoeNaponelli, ValeriaCaarls, LotteNarayanan, AnandCaballero-Garcia, BeatrizNardelli, CarmelaCabanillas, BeatrizNariai, TadashiCabral, HoracioNarikawa, ReiCabral, JaydeeNarimatsu, HirotoCabrele, ChiaraNarkar, Vihang A.Cabrera-Fuentes, H. A.Narute, PurushottamCaburet, SandrineNarváez, ManuelCaccamo, DanielaNascimento, KyriaCacciatore, IvanaNass, NorbertNúñez De Cáceres González, F. F.Nassuth, AnnetteCadamuro, MassimilianoNataf, SergeCádiz Gurrea, M. D. L. L.Natarajan, ArutselvanCaeiro, MariaNatarajan, Sathish KumarCaffarel, María MuñozNault, RanceCaffarelli, CarloNaumann, Todd A.Cafiero, MauricioNaumova, Anna V.Cahoon, Aubrey BruceNaumovski, NenadCai, DeminNavakauskiene, RutaCai, LuNavarro, Esther PérezCaiazzo, ElisabettaNaviglio, SilvioCairo, GaetanoNawaz, MuhammadCairrão, ElisaNazar, Ross N.Caivano, AntonellaNazaruk, JolantaCalabrese, Edward J.Neely, Brian J.Calado, ÂngeloNeergheen-Bhujun, VidushiCalder, PhilipNeilson, AndrewCaldera, FabrizioNejad, MojganCalì, TitoNelson, Fred R. T.Call, MindyNepal, MadhavCallaghan, BridNeubauer, Bernd A.Callendret, BenoitNeuhaus, JochenCalorini, LidoNeumann, JakeCalvano, Cosima D.Neureiter, DanielCalvo-Guirado, José LuisNeutelings, GodfreyCalzetta, LuiginoNevšímalová, SoňaCalzone, LaurenceNeymotin, FlorenceCamacho-Cristóbal, Juan J.Ng, DominicCamiciottoli, GiannaNguyen, Andrew V.Camire, MaryNguyen, T. K. P.Camlin, Nicole J.Ni, HongminCammarata, KirkNi, WeimingCampara, MayaNicholson, Louise F. B.Campbell, BradleyNicolaides, Nicolas C.Campos, EricNicolaides, TheodoreCampos, NarcisoNicolas, SkuliCampuzano, OscarNicoll, RachelCañas, Rafael A.Niculita-Hirzel, HélèneCandiani, GabrieleNiederberger, EllenCândido, ElizabeteNiederman, Robert A.Cangkrama, MichaelNiedziela, TomaszCanini, AntonellaNielsen, Vance G.Cannavo, EldaNiemz, AngelikaCannone Falchetto, AugustoNiessen, Frank B.Cansby, EmmelieNieto, GemaCantalapiedra, Carlos P.Nieuwenhuizen, Niels J.Cantile, MonicaNigris, Filomena DeCao, RenzhiNijnik, AnastasiaCapdevila, MercèNikas, Spyros P.Capel, CarmenNikawa, TakeshiCappariello, AlfredoNikolaev, Sergey I.Caprari, PatriziaNikolakopoulou, Angeliki M.Carapelli, AntonioNilsson, GunnarCarare, Roxana OctaviaNilsson, PeterCarboni, LuciaNilsson, Ulf J.Cardoso, SusanaNishiguchi, MasamichiCardoso, Susana M.Nishimura, KaneyasuCariati, FedericaNishimura, NorihisaCarlisi, DanielaNishimura, TaisukeCarloni, VinicioNishiwaki, HisashiCarlsen, Hanne KrageNishiyama, AkiraCarmine, GuarinoNisnevitch, MarinaCarmody, RuaidhriNissen, JillianCarnevale, DanielaNistala, RaviCarol, PierreNitta, TakeshiCarraro, MauroNitulescu, George MihaiCarraro, SilviaNiu, GenhuaCarrasco-Pancorbo, AlegríaNobili, StefaniaCarregal-Romero, SusanaNocentini, GiuseppeCarretón, ElenaNociari, MarceloCarrier, AliceNodomi, SeishiroCarrubba, AlessandraNofer, Jerzy-RochCarson, Monica J.Noge, KojiCarter, ClaireNoghero, AlessioCarter, WayneNoguchi, Constance TomCartland, SianNoguchi, EishiCarulli, LuciaNoguchi, TakuyaCaruso, GiuseppeNoguera, DanCarvajal, MicaelaNohe, AnjaCarvalho, KatherineNøhr, Mark K.Casal, SusanaNomikos, TzortzisCasalino, ElisabettaNomura, MikaCASARINI, LivioNonami, AtsushiCascio, SandraNonappa, NonappaCasini, GiovanniNonhebel, Heather M.Casorati, GiuliaNookala, SubaCastaldi, AlessandraNoordermeer, DaanCastel, HélèneNørholm, Morten H. H.Castella, MariaNoris, EmanuelaCastellano, OrlandoNorman, TrevorCastiglioni, BiancaNorris, ErinCastle, BrianNorris, Gillian E.Castorina, AlessandroNorton, NadineCastren, EeroNosoudi, NasimCaswell, Clayton C.Novack, Gary D.Cattaneo, FabioNovak, RichardCatty, PatriceNovick, DanielaCaudron-Herger, MaiwenNovío, SilviaCauli, OmarNowak, RobertCava, ClaudiaNowicki, MarcinCavada, Benildo SousaNozu, KandaiCavalla, PaolaNúñez Garrote, José IgnacioCavallari, MarcoNunn, Alistair V. W.Cavani, LucianoNunomura, WataruCayot, NathalieNuthikattu, SaivageethiCeccarelli, SaraNuutila, KristoCeccherini Guicciardini, M. T.Nyström, KristinaCecchetti, SerenaO’neill, Catherine A.Cehofski, LasseObara, YutaroCelli, AnnaObermüller, NicholasCelli, GiovanaOcchialini, AlessandroCenci, ElioOchoa Corona, FranciscoCengotitabengoa, MonicaOcio, EnriqueCeranowicz, PiotrO’Connell, GraceCerchia, LauraOdenthal, MargareteCeresa, BrianOdum, NielsCeriotti, AldoOfford, JamesCesare Marincola, FlaminiaOgasawara, ToruCesselli, DanielaOgata, TadanoriChahrour, MariaOgi, KazuhiroChak, KayamOgino, ShujiChakrapani, AnupamOgoh, ShigehikoChallacombe, Jean F.Ogunjirin, AdebowaleChallagundla, Kishore B.Oh, KeimeiChallet, EtienneOh, SeikwanChamcheu, Jean ChristopherOh, Yoon SinChami, BelalO’Hagan, Heather M.Chan, Ben C. L.O’Hara, StevenChan, Chi HoOhe, KenjiChan, Hue SunOhkohchi, NobuhiroChan, JulieOhmura, KoichiroChan, WanOhnishi, KohtaChand, SourabhOhnishi, MasatoshiChandaka, Giri KumarOhno, ShoChang, Chien-ChungÖhrfelt, AnnikaChang, Chuang-RungOhta, YukoChang, Hui-huaOikawa, AkiraChang, Ing-FengOjika, MakotoChang, Jer-MingOjima, KoichiChang, JinhoOkada, MuneyoshiChang, JungshanOkada, TomoyoChang, K. C.Okamoto, KoichiChang, Kee-LungOkamoto, MasamiChang, Ko-TungOkamoto, TakayukiChang, Long-SenOkino, TatsufumiChang, Wei-JenOklešťková, JanaChang, Wen-ChiOkochi, MinaChang, Wen-WeiOkumura, NobuoChao, Louis KuopingOkumura, TadayoshiChao, Wei-TingOlcese, James M.Chapel, AlainOlejar, Kenneth J.Chapman, Mark AOliveira, Paula AlexandraChapoval, Svetlana P.Oliveira, RaquelChari, SudarshanOliveira, RuiCharles, Luenda E.Oliveira-Ferrer, LeticiaCharlier, EdithOliver, BrianCharlton, ClivelOliver, RichardChass, Gregory AdamOlivier, ElodieChater, CasparOlivier, GiresChatterjee, JhinukOlofsson Bagge, RogerChatterjee, RatnaOlsen, NancyChatzigeorgiou, AntoniosOlson, Michael W.Chaudhuri, NaziaOltra, ElisaChaurand, PierreOmar, Syed HarisChaves-Martinez, F. J.Omranian, NooshinChaves-Pozo, ElenaOng, Eng ShiCheca, AntonioOnyenwoke, Rob U.Chegou, Novel N.Oon, Hazel H.Chelbi, Sonia T.Oosterwijk, EgbertChelmonska-Soyta, AnnaOosting, JanChemat, FaridOpazo, FelipeChen, Bor-SenOppert, BrendaChen, ChanghanOppido, Piero AndreaChen, ChiachungOprian, DanielChen, Chun-ChiOrban, LaszloChen, Chun-JungOrdas, BernardoChen, Chun-LinOrecchio, SantinoChen, Gen-HungOrecchioni, MarcoChen, GuoxunOrengo, DorcasChen, HongweiOrlova, Elena V.Chen, Hui-WenOrnstein, Moshe C.Chen, Jiann-ChuOrosa, BeatrizChen, JianwuOrozco, LuisChen, JiaoOrtega, NatividadChen, Kai-YiOrth, James D.Chen, KevinOrtolano, SaidaChen, Kun-MingOry, StéphaneChen, Lei-ShihOrynbayeva, ZulfiyaChen, LinyiOrzechowski, ArkadiuszChen, Miaw-LingOsborn, MarkChen, Min-HueyO’shaughnessy, K. M.Chen, Po-YuanOshima, YoshimiChen, Rong-JaneOshima, YusukeChen, Ruei-mingOshiumi, HiroyukiChen, SuzieOsier, NicoleChen, TianbaoOsman, NarinChen, TongOsolodkin, DmitryChen, Tzong-YuehOsorio, E. YanethChen, WayneOsorio, JohanChen, Wei-YuOsses, NelsonChen, Yei-TsungOtani, KeisukeChen, Yeong-RenOthumpangat, SreekumarChen, YiliangO’Toole, Sharon A.Chen, Yu-ChihOtsuka, FuminoriChen, Zhe-Sheng (Jason)Otsuka, YuzuruChen, ZhihangOuazzani, JamalChen, ZhijieOury, CécileChen, ZhonghuaOwen, ShawnChénais, BenoîtOyama, FumitakaCheng, JiaOyoshi, TakanoriCheng, Juei-TangOzaita Mintegui, AndrésCheng, XinlaiOzaki, IwataCheng, Yu-JungOzaki, ToshinoriChengwen, SunÖzalp, V. C.Cheon, Choong-illOzeki, YasuhiroCherkaoui Malki, MustaphaOzkok, AbdullahCherniack, Evan PaulOzsvari, BelaCherry, JonathanPace, Betty S.Chi, Chia-YuPacella, ElenaChi, Ching-ShiangPacheco, MárioChiadò, AlessandroPaci, MaurizioChiang, Hsiu-MeiPacios, Luis F.Chiang, Ming-KoPaduch, RomanChiang, Tai-anPadula, AndrewChiaraluce, RobertaPadula, MatthewChiariello, MarioPae, Eung-KwonChiba, YoshihikoPaek, AndrewChidambareswarapattar, ChakkaravarthyPagani, FrancoChien, ChihyenPagani, StefaniaChien, Hung-YuPagano, LucaChiessi, EsterPagano, MariaChilian, William M.Paggetti, JérômeChilosi, MarcoPaggiaro, PierluigiChim, StephenPagni, FabioChiou, Ya-LingPaik, JisunChirumbolo, SalvatorePaik, Yong-HanChitarra, WalterPain, BertrandChiu, Ka Fung PeterPaíno, TeresaChiu, Tsai-HsinPaiva, Cintia S. DeChlichlia, KaterinaPaja̧ķ, PaulinaChmurzynska, AgataPal, RiturajCho, Dong-WooPalacios, OscarCho, Joo-YounPalanisamy, Satheesh KumarCho, Seung HakPalei, AnaCho, Ssang-GooPalese, PeterCho, Yoon HeePalitti, FabrizioChobot, VladimirPaliyath, GopinadhanChoi, Dae EunPallotta, IsabellaChoi, Huimahn A.Palmero, DanielChoi, Je-MinPalmieri, DarioChoi, Je-YongPalmioli, AlessandroChoi, Seong-HoPalumbo, CarlaChoi, WoonyoungPaluzzi, Jean-Paul V.Cholkar, KishorePan, Hung-ChuanChong, Lee-GauPan, MiaoChou, Cheng-YangPanagiotidis, MihalisChou, Kuo-ChenPanagiotopoulos, Ioannis A.Choudhary, SanjeevPanaro, Maria AntoniettaChoudhury, DevasmitaPandey, Avinash ChandraChoudhury, MahuaPandey, Girdhar K.Choudhury, MalayPandita, TejChouker, AlexanderPanfoli, IsabellaChowdhry, SudhirPang, JinjiangChrist, TorstenPani, BibhusitaChristensen, BrockPanicker, RajeshChristians, ElisabethPanico, AntonioChristians, JulianPanov, KonstantinChristians, Kathleen K.Panzella, LuciaChristodoulou, Maria-IoannaPaoli, PaoloChristoffersen, MettePapaccio, GianpaoloChristopher W., KirbyPapadimitriou, LamprosChruszcz, MaksymilianPapadimitriou, Sofia A.Chrzanowski, ŁukaszPapadimitriuou, K.Chu, ShidongPapaioannou, DionissiosChua, Anita C. G.Papaleo, FrancescoChua, MelvinPapantonis, ArgyrisChuang, Lea-YeaPapazafiropoulou, A. K.Chuang, Yung-JenPapenfort, KaiChüeh, Anderly C.Papet, IsabelleChui, HelenaPapetti, AdeleChun, Heung JaePapi, AlessioChun, Kyung-SooPapini, AlessioChung, Byung MinPapp, AndrásChung, DonghoonPappalardo, DanielaChung, JayongParadowska-Gorycka, AgnieszkaChung, Jong HoonParashar, DeepakChung, SungjinParaskevis, DimitriosChyau, Charng-CherngParayath, Neha N.Ciaccio, MarcelloParcells, MarkCiancetta, AntonellaPardal, RicardoCiapetti, GabrielaPardigon, NathalieCiavattini, AndreaParekh, DhruvCicala, MichelePARINE, NARASIMHA REDDYCicciu, MarcoParisi, CristianCiccone, Marco MatteoParisini, EmilioCicero, ArrigoPark, ByungCichero, ElenaPark, ChankyuCichorek, MiroslawaPark, Hae RyounCignarella, AndreaPark, Hyun-WooCimmino, AmeliaPark, JoshuaCindrova-Davies, TerezaPark, JunsooCinelli, PatriziaPark, MaiyonCiobica, AlinPark, Sam-YongCipolla, DavidPark, Sung-GyooCippitelli, MarcoParker, CarolineCirilli, MarcoParker, Tony JCirillo, EmiliaParnetti, LucillaCisowsky, JaroslawParola, A. JorgeCitro, ValeParrish, AlanClark, Michelle A.Parsons, Jason L.Clarkson, AndrewPartch, Carrie L.Clausen, FrederikParthasarathy, SrinivasCleary, Margot P.Parveen, IffatClément, YvesParys, Jan B.Clementi, NicolaPascale, AlbertoCleren, CarinePascale, RosaClericuzio, MarcoPascarelli, Nicola AntonioClevenger, JoshPascu, IrinaCloutier, ConradPasini, LuigiCoan, P. M.Paslakis, GeorgiosCobley, JamesPasqualoto, KerlyCoccheri, SergioPassamonti, SabinaCocco, CristinaPassarinha, L. A. P.Cocetta, GiacomoPastor, FernandoCochran, BlakePastor, JesúsCohen, DanPastores, Gregory M.Cohick, WendiePasz, Lidia SasCohn, MartinPaszkiewicz, SandraColecchia, DavidPatel, Daxesh P.Coleman, William B.Patenaude, MathewColey, Helen M.Pathakoti, KavithaCollett, AndrewPatil, Naeem K.Collier, JasonPatil, Rajkumar V.Collin, RobPatrice, ThierryCollins, Maurice N.Pattekari, PravinCologna, StephaniePatterson, JenniferColombi, MarinaPaul, AntoniColombo, EmanuelaPaul, MatthewColonna, GiovanniPaulus, WernerColquhoun, Thomas A.Pavagadhi, ShrutiColussi, GianLucaPavankumar, TheethaComai, StefanoPavlidis, IoannisComfort, K. K.Pavlik, Edward J.Commisso, MauroPavlov, TengisCondorelli, Daniele FilippoPawar, SnehalataConese, MassimoPawełkowicz, M. E.Conlon, John MichaelPawlak, AgnieszkaConnaughton, Victoria P.Payne, AnnetteConti, HeatherPazdro, RobertConti, LucioPeana, Alessandra T.Contino, MarialessandraPeana, Massimiliano F.Contreras-Moreira, BrunoPearson, MarkCooke, Ira R.Pechanova, OlgaCooke, MarcusPécheur, Eve-IsabelleCook-Mills, Joan MPedersen, AndersCoppedè, FabioPedeux, RémyCoppotelli, GiuseppePedotti, RosettaCorbetta, SabrinaPedraza-Chaverri, JoséCordo Russo, Rosalia I.Peeples, MarkCordoba-Diaz, D.Peffers, Mandy J.Corella, DoloresPegolo, SaraCorniola, RikkiPeijnenburg, WillieCorreia-de-Sá, PauloPeinado, Juan R.Cortés-Castell, ErnestoPeiris, MadushaCortese, KatiaPeitzsch, ClaudiaCortez, Maria AngelicaPejin, BorisCosta, Maria ManuelaPekin, DenizCostantini, SusanPelagalli, AlessandraCostanzo, FrancescoPellegrini, Gretel G.Costa-Rodrigues, JoãoPelletier, GuillaumeCotella, DiegoPellis, AlessandroCotman, SusanPelosi, PaoloCourville, Elizabeth L.Peluso, GianfrancoCousin, XavierPeluso, IlariaCoutinho, João PauloPemov, AlexanderCoutinho, TeresaPeña, Maria Marjorette O.Covello, GiuseppinaPendyala, GuruduttCovès, JacquesPenfornis, PatriceCowell, IanPeng, Chien-fangCoyle, BethPeng, LifengCoyle, Patricia KPeng, RobertCraggs, Timothy D.Peng, TengCran, Marlene J.Peng, Wen-HuangCrawford, Bryan D.Penissi, Alicia B.Crawford, GemmaPenn, LindaCreamer, RebeccaPennington, KathleenCrescenzi, AnnaPennock, NathanCrescioli, ClaraPennypacker, KeithCrespan, EmmanuelePensabene, VirginiaCrespi, MartinPeracaula, RosaCrespo, Mariano SánchezPerego, CarlaCroci, DiegoPereira, C.Croset, MartinePereira, DavidCross, Carroll E.Pereira, Joana L.Crotti, TaniaPerera, OmaththageCruz, CeliaPerera, RushikaCruz-Fuentes, Carlos S.Perez, Juan J.Csabafi, KrisztinaPérez-Camino, Mari CarmenCsányi, GáborPérez-Castrillón, José L.Cubellis, MariaPerez-Gracia, TeresaCubero, Francisco JavierPérez-Pérez, José ManuelCucchiarini, MagaliPerez-Stable, CarlosCudalbu, CristinaPergament, EugeneCuesta, AlbertoPeris, KettyCui, HaitaoPerrais, MichaëlCui, XiaoyingPerreault, JonathanCui, YiPerrella, GiorgioCullen, CarliePerrine-Walker, Francine M.Culotta, Valeria CizewskiPerrotti, NicolaCulty, MartinePerry, Christopher G. R.Cuneo, Matthew J.Perticone, FrancescoCunningham, MichaelPes, Giovanni MarioCunningham, PatrickPešić, MilicaCurci, Pasquale LucaPesquet, EdouardCurtis, NicholasPeterbauer, Clemens KarlCusanelli, EmilioPetering, DavidCzabanka, MarcusPeters, Eva Milena JohanneCzarnocka, WeronikaPetersen, BjörnCzeglédi, LeventePeterson, JuliaCzerwinska, MonikaPetit, Patrice X.Czikora, IstvanPetrescu, Anca D.Czubryt, Michael P.Petrini, StefanoDa Silva Filho, Ademar AlvesPetropoulos, IsabelleDabrowiak, JamesPetrova, Olga E.Dacheux, Jean-LouisPetruczynik, AnnaDaftarian, PirouzPetrzik, KarelDai, Yang DPetta, IoannaDai, YunPettit, AllisonDailianis, StefanosPetukh, MarharytaDaina, AntoinePezzuto, AldoDale, RussellPhatarpekar, PrasadD’Alessandro, AngeloPhelps, Patricia E.Dalli, JesmondPhilip, MolyneauxDamaraju, SambasivaraoPhilipp, StephanDamjanovski, SashkoPhilips, NeenaDa’na, EnshirahPhillips, JacquelineD’Andrea, AntonioPhipps, SimonD’Angiolella, VincenzoPicardi, ErnestoDaniel, Marie-ChristinePicardo, MauroDanila, Daniel C.Picariello, GianlucaDanilenko, MichaelPiccoli, G. B.Dansette, Patrick M.PICOT, CedricDarby, IanPicozzi, MarioD’Argenio, ValeriaPiedade, Ana PaulaDark, Michael J.Piedra, Pedro A.Darwish, OmarPiiper, AlbrechtDas, AninditaPilati, StefaniaDas, LalitenduPilaz, Louis-JanDas, UndurtiPillay, VinessDas, ViswanathPilon-Thomas, ShariDasgupta, SubhamPilot, GuillaumeDatta, RupsaPina, MelanieDaugrois, Jean-HeinrichPina, SandraD’Auria, Maria ValeriaPineda, Asun MonfortDave, SandeepPineda, MiguelDaver, NavalPinho, Brígida RibeiroDaverey, AmitaPintea, AdelaDavide, FrancomanoPinto, AntonioDavidson, CristinPinto, Diana C. G. A.Davidson, DougPinto, MafaldaDavies, Jeffrey S.Pinton, PaoloDavis, FrankPinzani, PamelaDavis, Michael E.Piomboni, PaolaDavis, Michael J.Piplani, HonitDawn, ArnabPires, CarlaDawood, Mahmoud A. O.Pirici, DanielDe Aberasturi, D. J.Pirillo, AngelaDe Almeida Coelho De Sousa, Maria JoãoPiróg, Katarzyna A.De Amicis, FrancescaPiskuła, Mariusz K.De Andrés Cara, Damián F.Pisoschi, Aurelia MagdalenaDe Aza, PiedadPistone, AlessandroDe Berardis, DomenicoPitt, SamanthaDe Biase, DarioPitto, LetiziaDe Boer, M.K. (Marrit)Piva, TerrenceDe Bortoli, MichelePivac, NelaDe Brevern, AlexandrePiwowar, AgnieszkaDe Bruyne, ElkePizzolanti, GiuseppeDe Deurwaerdère, PhilippePla, DaviniaDe Feo, VincenzoPlacido, JerssonDe Francesco, FrancescoPlantier, LaurentDe Geest, BartPlarre, RuedigerDe Gonzalo, GonzaloPlatania, ChiaraDe Gonzalo-Calvo, DavidPlaté, ManuelaDe Graaf, PetraPlaza-Díaz, JulioDe Jong, Jill L.O.Plaza-Zabala, AinhoaDe La Cruz, José PedroPlażuk, DamianDe La Fuente, HortensiaPlazzi, FedericoDe La Garza, MireyaPleskot, RomanDe La Luz Garcia-Hernandez, MariaPluquet, OlivierDe La Monte, SuzannePniewski, TomaszDe La Serrana, Daniel GarciaPocheć, EwaDe Lago, EvaPodechard, NormandDe Lange, PieterPoeggeler, Burkhard H.De Lourdes Pereira, MariaPoggi, AlessandroDe Luca, LucianaPognonec, PhilippeDe Maagd, RuudPoidatz, DorothéeDe Martin, RainerPoitz, David M.De Mattei, MonicaPokora, WojciechDe Miguel-Beriain, IñigoPokrywka, AndrzejDe Paepe, BoelPolacco, Joseph C.De Paolis, AngeloPolaina, JulioDe Pergola, GiovanniPolakovičová, MájaDe Re, ValliPoldermans, DonDe Rosa, Maria CristinaPoleszak, EwaDe Rossi, AnitaPoltronieri, PalmiroDe Spiegelaere, WardPompili, MaurizioDe Vaufleury, AnnettePonomarenko, JuliaDe Vos, JohnPonomarev, EugeneDe Vrieze, ErikPonsaerts, PeterDe Wever, OlivierPoole, KateDe Yoreo, JimPooler, DarcyDe, KuntalPop, OanaDean, AfshanPopa-Wagner, AurelDean, JustinPopović Đorđević, JelenaDeb, SubrataPorebski, GrzegorzDeCicco-Skinner, KatiePorter, CraigDedecker, PeterPortis, EzioDeevska, GerganaPoschenrieder, CharlotteDegano, IlariaPossidente, BernardDegrassi, FrancescaPotenza, NicolettaDei Giudici, SilviaPotiron, VincentDe-Juan-Pardo, Elena M.Poulou, MyrtoDekaban, Gregory A.Powell, Ann L. T.Dekker, MarloesPowell, DanielDel Gaudio, CostantinoPowell, JoshuaDel Hierro, IsabelPowers, JenniferDel Mistro, AnnarosaPozo, Óscar J.Del Portillo, ArmandoPrado, RaquelDel Pozo, OlgaPrante, OlafDel Re, BrunellaPrasad, Asuri N.Del Real, GustavoPrats, Anne-catherineDelannoy, PhilippePreciado, DiegoDeleidi, MichelaPreissner, Klaus T.Delhanty, Patric J. D.Prensner, JohnDelihas, NicholasPrentice, HowardD’elios, Mario M.Prieto, IsabelDell’Agli, MarioPrieto-Lloret, JesusDellis, OlivierPrimiceri, ElisabettaDelogu, GiovannaPrince, JoseDelorme, VincentPrinsi, BhaktiDelporte, ChristinePrinz, Jörg C.Demir, FatihProctor, CaroleDemir, Ihsan EkinProestos, CharalamposDemyanets, SvitlanaProfessor Siu, Ming FaiDen Hartigh, Laura J.Profirovic, JasminaDeng, PengProft, ThomasDeng, QingProfumo, ElisabettaDeng, XufangProhens, JaimeDeng, ZhanaoProkai, LaszloDenizot, BenoitProost, PaulDenovan-Wright, Eileen M.Proudfoot, Amanda E. I.Deriu, Marco A.Pruett, DrewDeRosa, MariaPruna, AlinaDesai, AbhishekPrzekora, AgataDesai, JigarPrzepiera-Będzak, HannaDesando, GiovannaPrzyborowski, JerzyDeshmukh, RupeshPsarra, Anna-Maria G.D’Este, MatteoPu, JingDettweiler, UlrichPucciarelli, SandraDevan, Bryan D.Puccio, AvaDevasundaram, SanthiPuchta, HolgerDevesa, JesusPugh, NicholasDevine, Patrick J.Puiggalí, J.Devriendt, BertPulignani, SilviaDevy, JérômePulinilkunnil, ThomasDewitte, HeleenPuri, AkshitDhanasekaran, Danny N.Püschel, Gerhard P.Dhanasekaran, Saravana M.Pushkar, YuliaDhoot, G. K.Pusztaszeri, MarcDi Daniele, NicolaPutz, MihaiDi Fazio, PietroPuvvula, Pavan KumarDi Giacomo, SilviaPytka, KarolinaDi Guglielmo, Gianni M.Qi, DakeDi Liegro, ItaliaQi, Robert Z.Di Marco, StefanoQi, RongsuDi Masi, AlessandraQi, XinDi Nardo, MatteoQian, HongDi Primo, CarmeloQian, LiDi Renzo, LQian, PengxuDiack, Abigail B.Qin, GangjianDiana, PatriziaQin, HelenaDiapari, MarwanQin, HongweiDíaz, IsabelQuattrocelli, MattiaDiaz, Jose AntonioQuémerais, BernadetteDíaz, José FernandoQuesada Pérez, Víctor ManuelDickson, SuzanneQuigley, EamonDidonna, AlessandroQuillard, ThibautDiekman, AlanQuintana, JoséDiermeier, SarahQuock, RyanDierssen, MaraQvit, NirDíez, DavidRabbani, NailaDijkhuizen, LubbertRabien, AnjaDijkstra, GerardRabouille, CatherineDikalov, SergeyRachek, LyudmilaDimitrakopoulou-Strauss, A.Rachmawati, DessyDimitrios, StagosRadeke, Monte J.Dimitrov, Kiril M.Radenkovic, MiroslavDimova, ElitsaRafikova, OlgaDing, QiangRaftery, RosanneDing, Wen-XingRaggi, LorenzoDings, Ruud P.M.Ragusa, Maria AntoniettaDinh, Dzung H.Rahe, Michael C.Dini, LucianaRahimi, FaridDinischiotu, AncaRahman, MilladurDiniz, CarmenRai, PrakashDioum, Elhadji M.Raimondi, LaviniaDistel, MartinRaimondo, StefaniaDittmer, Neal T.Rainer, Peter PDitzel, MarkRainero, ElenaDixon, Ian M.C.Raingeaud, JoëlDmitriev, OlegRaj, Madhwa H. G.Do Canto, JavierRajaram, MurugesanDo, Sun HeeRajendran, Ranjith KumarDobens, Leonard L.Rajendran, VazhaikkurichiDobrowolska, GrażynaRajgor, DipenDocheva, DenitsaRajnarayanan, RajendramDoddapaneni, RaviRajpurohit, SurendraDoekes, GertRaju, JayadevDoi, KazuyaRakers, ChristinDoisneau-Sixou, SophieRakitin, AlekseiDolei, AntoninaRalli, MassimoDolinsky, Vernon W.Rallis, MicaelDolo, VincenzaRam Kumar, Ram MohanDomingos, Pedro M.Ramalingam, NaveenDomingues, FernandaRamalingam, VetrivelanDomingues, Renan B.Ramani, KomalDomínguez-Álvarez, EnriqueRamani, VijayDomozych, David S.Ramaraj, PanduranganDonadio, CarloRamasamy, SrinivasanDonato, RosarioRamchandani, DivyaDong, FengRamil, CarloDong, MingdongRamírez, Daniel RobledoDong, ZhengRamírez, VíctorDonnelly, OliverRamonda, RobertaDonnini, SandraRamos, SoniaDonohue, KevinRampias, TheodorosDonzelli, ElisabettaRamsahoye, BernardDores, Michael R.Ramsay, AlanDorfman, DamianRamsbottom, SimonDormond, OlivierRamshaw, HayleyDorrance, Anne M.Ran, ChongzhaoD’Orsi, BeatriceRana, DipakDosoky, Noura S.Randall, Stephen K.Dossena, SilviaRangachari, ManuDoulgeraki, AgapiRanjan, AlokDoumbia, SeydouRanjit, SumanDouros, Jonathan D.Ranney, Thomas G.Dovinova, ImaRantamaki, TomiDovizio, MelaniaRanzato, EliaDoyle, SiamsaRao, C. V.Draeger, AnnetteRapacz, MarcinDraijer, RichardRaposio, EdoardoDrenjančević, InesRapp, AlexanderDrews, OliverRäsänen, Leena A.Driscoll, James JRasid, OrhanDrobizhev, MikhailRasinger, Josef DanielDrougard, AnneRaspanti, MarioDrysdale, ThomasRastegar, MojganDu, MinRatajewski, MarcinDuarte, FátimaRatcliffe, IanDubey, AshishRaterman, H. G.Dubey, RaminRathinasabapathy, A.Dubielecka, Patrycja M.Ratkowsky, David A.Dubos, ChristianRatovitski, EdwardDubrova, YuriRatz-Łyko, AnnaDucza, EszterRauch, BernhardDudás, JózsefRaupach, MichaelDudeja, PradeepRauz, SaaehaDudok, JeroenRavaioli, StefanoDuennwald, Martin LRavegnini, GloriaDuffy, Aaron M.Ravet, KarlDugé De Bernonville, ThomasRavuri, SudheerDuh, Pin-DerRawal, AdityaDuke, Stephen O.Rawashdeh, OliverDuman-Scheel, MollyRawel, HarshadraiDumic, JerkaRawlings, NeilDumke, RogerRay, JayashreeDumont, PatrickRay, UdayanDunbar, Gary L.Rayan, AnwarDunn, LouiseRaychaudhuri, Siba P.Duong, Hai M.Reader, Jocelyn C.Duong-Quy, SyReale, MarcellaDuperray, AlainRebmann, VeraDuque, PaulaRecchia, AlessandraDurazzo, AlessandraReddy, SakamuriDuressa, DechassaRedell, MicheleDurgo, KsenijaRedmer, TorbenDurlik, MagdalenaRedondo Muñoz, JavierDurnford, DionReese, R. NeilDuryee, Michael J.Reeve, AmyDusi, VeronicaReeve, VivienneDussert, StéphaneRegal, JeanDutta, ArijitRegalado-González, CarlosDutta, PalashReginato, Mauricio J.Duverger, EricRegnard, Jean-LucDvorakova-Hortova, KaterinaRehrig, Erin M.Dyr, JanReid, Christopher W.Dziedzic, ArkadiuszReidy, PaulDziga, DariuszReigosa, Manuel J.Dziubla, ThomasReilly, Karlyne M.E. Katerinopoulos, H.Reinach, Peter S.Easmon, JohnnyReinders, JoergEben, AstridReinders, YvonneEbo, DidierReis, HenningEbrahimi, K. H.Reiser, Peter J.Ebrahimi-Fakhari, DariusReisinger, NicoleEchevarria, MiriamReisman, Scott A.Eckardstein, ArnoldReljic, RajkoEckardt, KristinRelógio, AngelaEcke, ThorstenRemaley, Alan T.Eckhard, UlrichRemaut, KatrienEdamakanti, ChandrakanthRemesh, Soumya G.Eder, Iris E.Remuzzi, GiuseppeEder, KlausRen, JianguoEdin, Nina JeppesenRenaudineau, YvesEdinson, Yara-VarónRenes, JohanEdirisinghe, MohanRenfroe, MichaelEdwards, Joshua R.Renneckar, ScottEdwards, Marten JohnRenò, FilippoEferl, RobertRenovanz, MirjamEfird, JimmyRevelli, LucaEgashira, NobuakiRevilla, PedroEgawa, GyoheiReymond, Jean-LouisEgerszegi, IstvánReynolds, Lindsay M.Eggeling, LotharRezaei-Ghaleh, NasrollahEggink, DirkRezvani, KhosrowEhlen, J. ChristopherRialland, MickaëlEhninger, ArminRiazi, SheilaEhrich, MarionRibeiro, EdnaEichhorn, LarsRibeiro, IsabelEide, DavidRibeiro, Maria Henriques L.Eidenberger, ThomasRibeiro-Barros, Ana I.Eiroa, José LuisRiccetti, LauraEl Midaoui, AdilRice, Lauren J.El Muayed, MalekRich, Jeremy N.El-Ali, AymanRicha, TambiElbaum, RivkaRichards, Renée StirlingElbayoumi, TamerRichardson, AlanElble, Randolph C.Richetto, JulietEldarov, M. A.Richly, HolgerEleftheriadis, TheodorosRicotti, LeonardoEl-Habr, Elias A.Rieder, MichaelElhanafi, Ahmad OmarRiediger, ThomasEl-Heliebi, AminRieger-Christ, Kimberly M.Elibox, WinstonRienzo, Valentina DiEliseev, Roman A.Riga, MariaEl-Kadi, SamerRigante, DonatoEl-kereamy, AshrafRigas, StamatisEllervik, ChristinaRigó, JánosElleuche, SkanderRijt, Leonie VanElli, StefanoRikke, NorregaardElliott, Paul R.Riley, David G.El-Matbouli, MansourRimbach, GeraldElse, KathrynRimoldi, LucaElshimali, Yahya I.Rimoldi, SimonaEmery, PeterRimondini, LiaEmiliani, CarlaRinnerthaler, GabrielEmoto, MakotoRinnerthaler, MarkEmpl, MichaelRisatti, GuillermoEmri, EszterRishi, ArunEndo, TatsuroRisk, Janet M.Engel, Paul C.Ristori, Giovanni P.Engelhart, AaronRivera, VictorEngh, Richard A.Rivero, FranciscoEngineer, Crystal T.Rivero, RosaEnguita, FranciscoRiveron, Jacob M.Enugutti, BalajiRivero-Pérez, M. DoloresEppler, ElisabethRizzo, Angela MariaErban, TomasRizzo, RobertaErdődi, FerencRizzolio, F.Erenpreisa, JekaterinaRizzuti, BrunoEri, RajRoan, Nadia R.Eritja, RamonRobert, EricErnst, Robert K.Robert, JeromeEscames, GermaineRobert, LeemaEscobar, CarolinaRobert-McComb, Jacalyn J.Eskandari, ArvinRobinson, CliveEspada, JesúsRobinson, KarenEspen, LucaRobles Ramos, PedroEspinosa-Diez, CristinaRobson, GeoffEsposito, CiroRobson, MatthewEsposito, DeboraRoccheri, Maria CarmelaEsposito, Emilio XavierRocha Ferreira, EridanEsposito, SergioRocha, EduardoEstaras, ConcepcionRocha, SoniaEsterio, MarcelaRochaix, Jean-davidEsteve-Romero, JosepRoche, Daniel BarryEstevez, Jorge D.Rödel, FranzEstevinho, LeticiaRodella, Luigi F.Etienne, BechtRodenbeck, Stacey DineenEtxebeste, OierRodius, FrançoisEuaggelos, GikasRodrigo, IsmaelEuler, GerhildRodrigo, María JesúsEvans, FranklinRodrigues, AlírioEvans, Gareth John OwenRodrigues, MelanieEvans, GethinRodrigues, NunoEvans, Joseph L.Rodrigues, OlivierEvans, MalkanthiRodríguez Romero, AdelaEve, David J.Rodriguez, AlejandroEverett, ThomasRodríguez, Alexander J.Ezendam, JanineRodriguez, ChristopheEzura, YoichiRodriguez-Aguayo, CristianF Wallace, DanielRodriguez-Algaba, JulianFabiani, R.Rodriguez-Lafrasse, ClaireFabris, LindaRodríguez-Llorente, I. D.Fabris, LucaRodriguez-Menocal, LuisFacchini, GaetanoRoe, R. MichaelFaehling, MartinRoeb, ElkeFaenza, IreneRoelen, BernardFairweather, StephenRoelofs, JeroenFaísca, PatríciaRoffi, AliceFalcieri, ElisabettaRogozhin, EugeneFalkinham, JosephRogozin, IgorFamiani, FrancoRoh, ChanghyunFan, BinRoh, SanghoFan, Hueng-ChuenRohira, AartiFan, WeiRokstad, Anne MariFan, ZhichaoRomagnoli, StefanoFaner, RosaRomani, MassimoFang, Hsu-WeiRomano, AndreaFang, RonnieRomano, NiclaFang, XiRomanov, VictorFang, XiaolanRomee, RizwanFang, YiRomei, CristinaFanizzi, Francesco P.Romero Martínez, ÁngelFantacuzzi, MarialuigiaRomero, AlejandroFantuzzi, LauraRomero, CarmenFaouzi, MalikaRomero-Sandoval, AlfonsoFareed, JawedRonca, AlfredoFaria, J.Roncada, PaolaFarnoud, AmirRonchetti, SimonaFarr, JoshuaRong, YueguangFarr, TracyRooj, ArunFarrar, MarkRoos, JessicaFarris, StefanoRoper, Randall J.Faruqi, Mohamed A.Roque, CláudioFasler-Kan, ElizavetaRos Lis, Jose VicenteFast, Loren D.Ros, Marian A.Faundez, VictorRosales-Mayor, EdmundoFaure, SébastienRose, GiuseppinaFava, PaoloRosellini, DanieleFederico, AntonioRosendahl, JonasFedorova, Olga V.Ross, IanFehrentz, Jean-AlainRoss, Ian L.Fei, PeiwenRossa Jr., CarlosFeinendegen, LudwigRossi, SimonaFelipe Farias, DaviRossignol, JulienFelix, Klaus M.Rossman, JeremyFelix, ManuelRossner, PavelFelling, RyanRostaing, LionelFeng, Liu-SmithRotllant, JosepFeng, WenyiRotomskis, RicardasFeng, YibinRoubelakis-Angelakis, K.Feng, YumeiRoubertoux, PierreFeng, Zhi ChaoRouhiainen, AriFenton, RobertRouse, Matthew N.Feo, FrancescoRoutledge, MichaelFeraco, AlessandraRouws, LucFergani, ChrysanthiRoviello, GiandomenicoFerguson, DavidRoy, DeoduttaFerlini, AlessandraRoy, JérômeFernández, IgnacioRoy, KasturiFernández, LucíaRoyo, FelixFernández, MartaRoz, LucaFernandez-Botran, RafaelRozanov, DmitriFernandez-Jimenez, NoraRozhon, WilfriedFernández-Ponce, M. TeresaRozot, VirginieFernández-Rodríguez, JavierRua, DiegoFernández-Ruiz, JavierRuan, XiangboFerrante, ClaudioRubino, Federico MariaFerreira, Leonardo G.Rubio, VicenteFerreira, Tracie L.Rubis, BlazejFerreon, JosephineRudd, SeanFerrera, ReneRuden, DouglasFerretti, ElisabettaRuderman, DanFerrio, J. P.Rudkin, BrianFerriol, InmaculadaRudrabhatla, Sairam V.Fettkenhauer, ChristianRudyk, OlenaFicai, AntonRuelland, EricFickert, PeterRuest, L. BrunoFiering, Steve N.Ruff, AdrianFigueiras, EditeRuiter, Godard C. W. DeFigueiredo, Marxa L.Ruiz, SergioFigueroa, Carlos R.Ruiz-Ederra, JavierFilek, MariaRuiz-Gómez, GloriaFilella, XavierRuiz-Lozano, Juan ManuelFilippova, MariaRuiz-Narváez, Edward A.Filleur, StephanieRund, DeborahFilosa, AldoRupenthal, IlvaFilosto, MassimilianoRuppert, J. MichaelFina, EmanuelaRupprecht, RainerFindlay, DavidRurek, MichalFine, James BurkeRusso, GiuliaFinelli, CarmineRusso, JoséFineschi, VittorioRusso, MariaFingerhut, RalphRusso, Matteo A.Finley, BrentRusso, PasqualeFinn, AlokeRusso, SuzanneFioravanti, AntonellaRuzicka, JiriFiorucci, StefanoRyffel, BernhardFirestein, Bonnie L.Rylott, Elizabeth LFisch, Gene S.Ryu, Jong HoonFisher, Aron B.Rzymowska, JolantaFitzHarris, GregSaarma, MartFixen, KathrynSacco, AngelaFleckenstein, JohannesSacco, EmilioFletcher, Nicola F.Saccone, ValentinaFlex, AndreaSachmechi, IssacFlieger, JolantaSachs, GeorgeFliniaux, OphélieSacitharan, Pradeep KumarFlorczyk, Stephen J.Sadoghi, PatrickFlorio, TullioSadowska, BeataFlorsheim, EstherSaelens, XavierFonseca, AnaSaera-Vila, AlfonsoFonseca, FilomenaSaez, CarmenFontalba-Navas, AndresSafa, AhmadFontana, LucaSah, Hong-KeeFontana, Robert J.Saha, AchintoFontes, PauloSahin, MustafaFoo, EloiseSaikumar, PothanaForcales, Sonia-V.Saini, ShikhaFord, HenriSainlos, MatthieuFormisano, LuigiSaisho, YoshifumiFormisano, PietroSaito, HideyukiForn-Cuni, GabrielSaito, YoshiroForneris, FedericoSaiz-Fernández, IñigoForostyak, SerhiySajic, MarijaForster, BrittaSakaguchi, YusukeForster, JamesonSakai, AtsushiForster, John WSakai, MasahiroFort, PatriceSakai, RyuichiFoster, JohnSakamoto, KeiFosu-Nyarko, JohnSakane, FumioFouassier, LauraSaki, NajmaldinFoubert, PhilippeSakuma, TetsushiFourtounas, CostasSala, GianlucaFox, GlenSaladino, RaffaeleFozo, ElizabethSalaspuro, MikkoFracasso, AlessandraSalazar, GloriaFrachisse, Jean-MarieSalbach-Hirsch, JulianeFradet-Turcotte, AmélieSalehin, MohammadFraifeld, Vadim E.Salek, RezaFrance, MichaelSalgueiro, Carlos A.Francisco González, JorgeSalim, VonnyFranco, A. C. R.Salio, MariolinaFranco, DiegoSaluk-Bijak, JoannaFranco, LuisSalvador-Morales, CarolinaFranco, Manuela DiSalvarani, Nicolo’Franco, RodrigoSalvati, BrunoFrancolini, IolandaSalvatore, MirellaFrancolini, MauraSalvi, SamantaFrangogiannis, NikolaosSamant, Rajeev S.Frank, SašaSamarakoon, RohanFrankel, Timothy L.Sampathi, ShilpaFranken, NicolaasSamuel, ChrishanFranklin, Renty B.Samuel, MarcusFranko, AndrasSan Martin, CarmenFrantom, PatrickSanak, MarekFranzè, AnnamariaSánchez, Beatriz JuradoFraser, Justin F.Sánchez-Pernaute, OlgaFrau, JuanSanders, Alison PFreeman, EdwardSands, Jeff M.Freeman, Jennifer L.Sangiolo, DarioFreeman, Linnea R.Sangiovanni, EnricoFrias, JuanaSangireddy, Sasikiran ReddyFrieboes, HermannSanij, ElaineFriedlander, Terence W.Sanina, NinaFroemke, Robert C.Sanjust, EnricoFrolov, AndrejSankaranarayanan, Nehru VijiFrolova, LiliyaSanner, Michel F.Fromm, BastianSanoh, SeigoFrotscher, MartinSansone, ClementinaFrugis, GiovannaSantamaría, EstrellaFu, JunjieSantamaria, RitaFu, Wen-MeiSantambrogio, PaoloFu, YongzhuSantander, JavierFuchs, DietmarSantander-Jiménez, SergioFujie, TomoyaSantato, ClaraFujihara, HisakoSanthakumar, AbishekFujii, TomomiSanthosh, Sebastin M.Fujimori, KoSantin, IzortzeFujita, MitsuguSantino, AngeloFujita, TetsujiSantolla, Maria FrancescaFujita, YuichiSantoni-Rugiu, EricFukuchi, SatoshiSantoro, CristinaFuller, BryanSantos, Maria JacintaFumagalli, FabioSantos-Gallego, CarlosFunaba, MasayukiSantourlidis, SimeonFunder, JohnSanz, IvanFunel, NiccolaSanz-Clemente, AntonioFurfaro, Anna LisaSaper, CliffordFurness, John B.Sarath, GautamFürnsinn, ClemensSarbini, ShahrulFurue, MasutakaSarkar, SujanFurukawa, ToruSarker, DipakFuruya, HidekiSarmast, Nima D.G., MusumeciSarniguet, AlainGabbianelli, RositaSarnowski, TomaszGaczynska, MariaSasaki, MasanoriGadaleta, MarianaSassi, MohamedGaddam, Ravinder ReddySassi, YassineGaddameedhi, ShobhanSatani, NikunjGadjeva, MihaelaSatbhai, SantoshGagos, SarantisSather, NoahGahete, Manuel D.Satish, LathaGahlaut, VijaySato, Masa H.Gaida, MatthiasSato, TakuichiGajendrareddy, Praveen K.Satoh, ShigeruGajula, RajendraSatou, RyousukeGál, PeterSatou, TadaakiGalanis, AlexSattar, SampurnaGalat, AndrzejSaturnino, CarmelaGaleazzi, RobertaSau, SamareshGaleffi, FrancescaSauka, DiegoGalek, RenataSaunders, NicholasGaletti, MariclaSaurav, KumarGalileo, Deni S.Savaskan, Nicolai E.Galisteo Jr., Andres JimenezSaville, RobertGall Troselj, KoraljkaSavion, NaphtaliGallardo, Rodrigo ASavoie, Jean-MichelGalli, Dominique M.Sawa, TeijiGallicchio, EmilioSawada, KenjiroGallo, LindaSawai, HirofumiGaluska, SebastianScafoglio, ClaudioGalve-Roperh, IsmaelScahill, LawrenceGambarotta, GiovannaScala, ValeriaGan, BoyiScandurra, RobertoGan, RenyouScanes, Colin G.Gan, Ren-YouSchachter, MichaelGangula, PanduSchaedler, Theresia A.Ganguly, AnutoshSchaefer, JeremyGanini, DouglasSchaefer, MichaelGantenbein, BenjaminSchafer, PeterGanz, JuliaSchäfer, WilhelmGao, DianSchaffner, WalterGao, YanzheSchallenberg-Rüdinger, M.Gao, YunxiangScharfman, HelenGarcía Hidalgo, JavierSchefold, Joerg C.Garcia Ivars, JorgeSchembri, MarkGarcia, Lorena CarroSchepens, BertGarcía-Escudero, RamónSchepisi, GiuseppeGarcia-Estañ, Luis PerezScherberich, ArnaudGarcia-Gonzalez, Carlos A.Scherer, GüntherGarcía-Martínez, EvaSchernthaner, Gerit-HolgerGarcía-Mauriño, SofíaSchettino, GiuseppeGarcía-Villalón, Angel LuisSchiavone, MarcoGardin, ChiaraSchiavone, StefaniaGariboldi, ManuelaSchildgen, OliverGarinis, GeorgeSchiller, DirkGarrett, ScottSchinke, ThorstenGarrido, DoloresSchirmer, BastianGarriga, PereSchisler, JonathanGarrosa, ManuelSchlaepfer, Isabel R.Gary-Bobo, MagaliSchlegel, JürgenGasche, ChristophSchlierf, MichaelGaspar, DianaSchlueter, Klaus-DieterGaspar, KrisztianSchmedake, Thomas A.Gáspár, RóbertSchmid, MarkusGasparrini, MassimilianoSchmid, MichaelGassler, NikolausSchmid, TobiasGassman, Natalie R.Schmidt, AnkeGaszner, BalázsSchmidt, MartinGatti, LauraSchmidt, Mirko H. H.Gaudenzio, NicolasSchmidt, TanninGaudin, RobertSchmidt-Arras, DirkGauger, Phillip C.Schmitt-Egenolf, MarcusGauthier, Patrick T.Schmutzhard, ErichGavin, David P.Schnapp, LynnGavira, JoséSchneider, Achim G.Gavrilovic, JelenaSchneider, AnjaGawarecka, KatarzynaSchneider, BarbaraGazerani, ParisaSchob, StefanGe, XiaodongScholz, SonjaGebel, ThomasScholz-Starke, JoachimGehrmann, MathiasSchomburg, LutzGeib, ScottSchonthal, AxelGeisler, SarahSchousboe, ArneGelmini, StefaniaSchrag, MatthewGenetos, DamianSchraufstatter, Ingrid U.Geng, FengSchroeder, DanaGentile, CarlaSchroit, AlanGentile, PiergiorgioSchubert, MarioGeorgakilas, AlexandrosSchuhmann, Michael K.George Parsons, K. S.Schulte, Jan SebastianGeorge, SophiaSchultz, LioraGeorge, VargheseSchulz, Benjamin L.George, VinojSchulz, RichardGeorgia, SentaSchulz, Wolfgang A.Georgiev, VasilSchulze, JohannesGeorgieva, RadostinaSchumacher, AnneGeraci, FabianaSchumacher, JuliaGerardi, CarmelaSchumann, CananGerdol, MarcoSchütte, KerstinGericke, MartinSchutte, Stacey C.Gerke, OkeSchuur, BoeloGerlach, JaredSchuurman, Henk-JanGermain, LucieSchwartz-Albiez, ReinhardGermain-Aubrey, CharlotteSchwarz, Richard I.Geso, MoshiSchwarz, Steven M.Gewirtz, David A.Schweiger, ThomasGhaleh, BijanSchweiger, WolfgangGhayour-Mobarhan, MajidSchweizer, MichaelGherzi, RobertoSchwiebs, AnjaGhosh, BiswarupScicchitano, PietroGhosh, SouravScipione, LuigiGhoshal, KalpanaScorziello, AntonellaGiambona, AntoninoScott, Helton W.Giamperi, LauraScotti, MarcusGiampieri, FrancescaScotto D’Abusco, AnnaGiannattasio, SergioScovassi, Anna IvanaGiannelli, GianluigiScrima, MarioGiannoni, PatriziaScudiero, OlgaGiansanti, FrancescoScudiero, RosariaGiaouris, EfstathiosSebastian, AimyGibbison, BenSeca, AnaGijs, MarliesSeca, Ana M. L.Gilaberte, YolandaSechi, GianPietroGilch, SabineSediva, AnnaGilding, EdwardSedmiková, MarkétaGilding, Edward K.See, ViolaineGiles, WaltersSeeliger, ClaudineGilli, FrancescaSeffens, William S.Gil-Mohapel, Joana M.Segall, Jeffrey E.Gilson, EricSegura, AnaGiordano, AntonioSehrawat, ArchanaGiosafatto, ValeriaSeifitokaldani, AliGiovanni, FranzoSeiler, Magdalene J.Giovarelli, MatteoSeimiya, HiroyukiGirolomoni, GiampieroSeiquer, IsabelGirouard, HélèneSeki, NaohikoGiuseppina, AndreottiSeki, YoshinobuGiust, DavideSekine, ShuichiGłąbska, DominikaSelby, KatjaGlading, Angela J.Seliger, CorinnaGlasser, Wolfgang G.Seligmann, HervéGlick, Benjamin S.Selim, SamehGloria, AntonioSella, LucaGnanamony, ManuSellix, Michael T.Gnanasekaran, AswiniSelmin, Ornella I.Gniewosz, MałgorzataSelsby, JoshuaGnocchi, DavideSemenov, Victor V.Gobbi, GabriellaSempere, LorenzoGobe, Glenda CSengupta, BidishaGockel, InesSengupta, DebrupGodfrey, Henry P.Senter, Peter D.Godschalk, RogerSeo, Cheong HoonGoedeke, LeighSeo, JungwonGohda, EiichiSerafini, GianlucaGoicoechea, NievesSergaki, Maria ChristinaGok, OzgulSergi, Consolato MariaGoldberg, Lynne J.Serpico, RosarioGoldhawk, DonnaSerra, DolorsGoldklang, Monica P.Serra, FranciscaGoldoni, MatteoServis, MichaelGoldring, MarySesaki, HiromiGoldsmith, David R.Seth, DevanshiGoldstein, DavidSethi, GautamGolenhofen, NikolaSetou, MitsutoshiGoletti, DeliaSetzer, WilliamGoltsov, AlexeySeva, CatherineGomes, Ana P.Sevcikova, SabinaGomes, Maria SaloméSeymour, NikkiGomes, Selma A.Sgarra, RiccardoGómez De Cedrón, MartaSha, ZheGómez Llorente, CarolinaShabbir, WaheedGomez, JordiShah, DilipGomez, NidiaShah, NilayGomez-Lopez, NardhyShahbazi, Mohammad AliGomez-Sanchez, CelsoShaikh, Mohamad GuftarGomis-Rüth, F. XavierShaikh, Mohd. FarooqGondhalekar, Ameya D.Shang, LiGondi, Christopher S.Shankar, EswarGondret, FlorenceShanmugam, MuruganandanGong, FadeShannon, OonaghGong, LiminShao, FangweiGong, Siew-gingShao, KanGonkowski, SlawomirShapiro, Allison L. B.Gonzales, Cara B.Sharma, AlokGonzalez Bosc, Laura V.Sharma, AnjaliGonzalez, AntonioSharma, Anurag L.González, Florenci V.Sharma, AtulGonzalez-Aguilar, GustavoSharma, BheshGonzalez-Barcala, F. J.Sharma, HimanshuGonzalez-conejero, RocioSharma, NaveenGonzález-González, RogelioSharma, RohitGonzález-Maeso, JavierSharma, SudhaGonzález-Miguel, JavierSharma, SushilGonzalez-Perez, Ruben R.Shaw, LisaGonzález-Vallinas, MargaritaShaw, OdetteGoppelt-Struebe, MargareteShecterle, Linda M.Goreham, ReneeSheen, Shyn-ShinGorgoulis, VassilisSheikh, NadeemGoriely, StanislasShelton, HollyGorini Da Veiga, AnaShemanko, CarrieGorman, AdrienneShemfe, MobolajiGórnaś, PawełShen, Che-Kun JamesGorodetsky, RaphaelShen, Szu-ChuanGorski, JeffreyShen, YangGortan Cappellari, GianlucaShen, ZhenyuGosselet, FabienSheppard, Mary N.Goswami, NanduSherbenou, DanielGotea, ValerSherrard, RachelGoua, MarieShertzer, Howard G.Goukassian, DavidShewale, SwapnilGouveia, SusanaShi, HuaGowans, EricShi, JiaqiGoyal, RajniShi, YunhuaGozzini, AntonellaShiao, Young-JiGrabrucker, Andreas M.Shigdar, SarahGradoni, LuigiShih, Yu-RuGraf, GregoryShiina, TakashiGraham, ReginaShim, Yhong-heeGraier, WolfgangShimada, TsutomuGräler, MarkusShimada, YasuhitoGralinski, LisaShimura, HanakoGramlich, MichaelShinoda, KeiGrammatikakis, IoannisShiozawa, YusukeGramzow, LydiaShipman, KateGranados-Principal, SergioShirahata, TatsuyaGrande, FedoraShiraishi, TakehikoGranja, AitorShiue, Yow-lingGranot, DavidShokal, UpasanaGrant, GeorgeSholkamy, EmanGrant, WilliamShostak, AntonGrãos, MárioShpak, Elena D.Grapputo, AlessandroShteinberg, MichalGrases, FelixShukla, Alpana P.Grassi, GabrieleShukla, GirishGray, ClintShukla, SanjeevGraziani, MaristellaShulman, AdrianGraziano, GuellaShysh, Angela M.Grazul-Bilska, AnnaSibilla, SaraGrazzi, LiciaSibille, Etienne L.Grbeša, DarkoSidari, RossanaGreene, Catherine M.Sidiropoulos, ChristosGreene, Lesley H.Sidorenko, Viktoriya S.Greene, Paul E.Sié, PierreGreenfield, JohnSieber, FritzGreenhalgh, DavidSiebuhr, A. S.Greenwood, Michael T.Sier, CornelisGregory, Richard L.Sierecki, EmmaGreither, ThomasSikkema-Raddatz, BirgitGrether-Beck, SusanneSilina, YuliyaGrewal, Raji PaulSilipo, AlbaGrewe, FelixSill, HeinzGrieco, DomenicoSilva, Gabriela JorgeGrieco, FrancescoSilva, Luís R.Grillari, JohannesSilva, Margarida F. B.Grimholt, UnniSilva, Nuno A.Grimm, MarcusSilva, Susana N.Grinberg, DanielSilva-Fisher, JessicaGrinfeld, JacobSilvestri, GiulianaGrishin, NickSilvestri, IdaGroh, Isabel Anna MariaSilvestri, RomanoGromadzka, KarolinaSilvestris, NicolaGrones, PeterSimes, DinaGrootaert, CharlotteSimko, FedorGros, FrédéricSimkó, MyrtillGrösch, SabineSimmons, Peter A.Gross, ChristinaSimões, Marta FilipaGross, Joshua B.Simon, Marie-ChristineGrosse, JirkaSimon, Sanford M.Grossi, GiancarloSimon, Scott I.Grossmann, Tom N.Simoni, JanGrothusen, ChristinaSimonsson, StinaGrotta, James C.Simpson, CraigGroveman, BradleySimpson, Julie E.Gruber, TanjaSinger, EstherGrumet, RebeccaSingh, AmareshwarGrzebelus, DariuszSingh, AnilGrzesiuk, ElżbietaSingh, AtulGu, FengSingh, Atul KumarGu, LiuqiSingh, BhagirathGu, ZhenSingh, Chandra K.Guan, BinSingh, ChanpreetGuardado-Calvo, PabloSingh, GuramritGubin, DenisSingh, JugpreetGuedens, WandaSingh, NishaGuéguen, LaurentSingh, RajbirGuerini, Franca R.Singh, RajeshGuerlesquin, FrancoiseSingh, SonalGuerriero, GeaSingh, Vijay K.Gugatschka, MarkusSingh, VishalGuha, Prajna P.Sinha, AmitGuha, SoniaSinha, KalyanGuhathakurta, SubhrangshuSiniscalco, DarioGuidotti, AlessandroSinn, MarianneGuignabert, ChristopheSiomi, HarukikoGuil, SòniaSipponen, Mika HenrikkiGuilpain, PhilippeSirdah, MahmoudGuimaraes, FernandoSirotkin, Alexander V.Gulati, PankajSisley, StephanieGulick, Patrick J.Sissung, Tristan M.Gundara, JustinSista, SubhashGunia-Krzyżak, AgnieszkaSita, GiuliaGuo, FenghaiSitaru, CassianGUO, PUSi-Tayeb, KarimGuo, Tai L.Sivakumar, SushamaGuo, WeiminSivasubramaniyan, KavithaGuo, Zong ShengSiwek, AgataGupta, AnkitSizochenko, NataliaGupta, SudhiranjanSkalko-Basnet, NatasaGupta, Vivek K.Skalova, LenkaGurha, PriyatanshSkendros, PanagiotisGurikov, PavelSkibicka, KarolinaGursoy, GamzeSkov, JeppeGustafsson, RasmusSkov, Per StahlGutiérrez, Raúl SánchezSkundric, Dusanka S.Gutiérrez-Juárez, RogerSlama-Schwok, AnnyGuzmán, Esther A.Slattery, DavidGwinn, William M.Slavik, RogerH Van Der Horst, EdwardSleutels, TomHa, SandieSlieker, Roderick C.Haase, HajoSloan, Kenneth B.Habiba, KhaledSłoczyńska, KarolinaHaboubi, NadimSlominski, AndrzejHachiya, TsuyoshiSlot, Jason C.Hack, EthanSlouka, ChristophHackenberg, StephanSlovin, SusanHackl, MatthiasSłupecka, MonikaHadjiargyrou, MichaelSmagghe, GuyHadjipavlou-Litina, DimitraSmani, YounesHadler-Olsen, ElinSmarr, Benjamin L.Haefliger, SimonSmart, NeilHaenen, GuidoSmartt, Chelsea T.Hagenlocher, YvonneSmed-Sörensen, AnnaHagerman, Randi J.Smeets, Eric E.Hagmann, SébastienSmekalova, Elena M.Hait, NitaiSmeriglio, AntonellaHallberg, BengtŚmieszek, AgnieszkaHall-Mendelin, SonjaSmirnovas, VytautasHamada, FumikaSmith, Brittany L.Hamaguchi, MasahideSmith, Ellen M. LavoieHamanishi, JunzoSmith, Ernest E.Hamann, MartineSmith, G. M.Hammer, SuntreaSmith, JessicaHammers, Christoph M.Smith, MarkHammerschlag, Margaret R.Smith, Micholas DeanHampel, DanielaSmith, Stephen M.Han, Dong WookSmith, Tim A. D.Han, JaehongSmutzer, GregoryHanai, Jun-ichiSmýkal, PetrHanaoka, MitsumasaSmyth, Lesley A.Hancock, RobertSneddon, JenniferHane, JamesSneyd, Mary JaneHanke, TomášSoares, Claudio M.Hannibal, LucianaSoenen, Stefaan J.HANO, ChristopheSohl, ChristalHanover, D John A.Sohn, Young ChangHans, GuySokolova, ViktoriyaHanschen, Franziska S.Solà Alberich, RosaHanson, PeterSolano, FranciscoHanss, MichelSoldevilla, MarioHanus-Fajerska, EwaSoleri, DanielaHaque, InamulSoler-Vasco, LauraHaque, RashidulSolomon, MatiaHara, AkiraSolorio, LuisHarada, MamoruSon, Deok-sooHarada, ShukoSon, Mi-WonHarashima, NanaeSong, ChunhuaHardeland, RudigerSong, JeongminHardingham, Jennifer E.Song, JinhuaHardy, John G.Song, MinseokHariharan, SeethalakshmiSong, SuHaro, DiegoSonkoly, EniköHarper, AlanSonnier, RodolpheHarris, Randall E.Sonntag, Kai-christianHarris, WilliamSonsalla, Patricia K.Harrison, Jonathan S.Soole, KathleenHarrison, MarkSoopramanien, AnbaHar-Shai, YaronSorriento, DanielaHart, MartinSosa, SilvioHartshorn, KevanSosna, JustynaHarvey, AdamSosne, GabrielHarvey, RuthSotillo, JavierHas, CristinaSoudeyns, HugoHasanuzzaman, MirzaSoulika, AthenaHasegawa, MidoriSounni, Nor EddineHasegawa, MorifumiSousa, Célia T.Hasenbein, SimoneSousounis, KonstantinosHashimoto, MakotoSouza, Glauco R.Hashimoto, NaotoSouza, Rhonda FrancesHassan, ImanSoveral, GraçaHassan, MohamedSoverini, SimonaHassan, Sherif T. S.Spanakis, MariosHassouna, RimSpandou, EvangeliaHata, AaronSpano, GiuseppeHatano, TsutomuSparacino-Watkins, CourtneyHatfield, Ronald D.Spassov, SashkoHatfield, Stephen M.Spelman, LyndaHattori, YuichiSpengler Neff, AnetHatzis, PantelisSpengler, DietmarHaubner, FrankSperling, Mark A.Hauser, Michael ArthurSpetz, JohanHausman, G. J.Spickett, CorinneHavemeyer, AntjeSpiegel, MartinHaverkamp, Richard G.Spiewak, RadoslawHaworth, OliverSpitzner, MelanieHayakawa, TohruSpoerl, DavidHayakawa, YoshihiroSpring, KevinHayashida, MorihiroSpyridopoulos, IoakimHayer-Hartl, ManajitSreedasyam, AvinashHayes, AlanSreedhar, RemyaHayes, SidneySreerama, SubramanyaHaynes, DavidSridhar, JayalakshmiHaynes, ErinSrihari, SriganeshHaynes, Kathleen GSrivastava, AkhilHazak, OraSrivatsan, Eri S.Hazane-Puch, FlorenceSrivatsan, MalathiHe, FeiSrivenugopal, KalkunteHe, HongliangSt John, James A.He, KangminStacchiotti, AlessandraHe, PeijianStacey, M.Heaney, J. L.Stadler, MarcHeaphy, ChristopherStafford, PhilipHearn, Michael J.Stagos, DimitriosHebbard, LionelStåhle, MonaHeber, DavidStaines, Katherine AnnHeberden, ChristineStallcup, WilliamHedges, JodiStambuk, NikolaHeery, David M.Stančiaková, LuciaHefferon, KathleenStancu, Andreea LuciaHegedűsová, AlžbetaStandage, Daniel S.Hegyi, EszterStaneva, DesislavaHehlgans, StephanieStanford, JohnHeidari, MohammadStaniszewska, MonikaHeidorn-Czarna, MalgorzataStanley, JoneHeiker, John T.Stanton, Robert C.Heimburg-Molinaro, JamieStarostik, PetrHelfield, Brandon L.Staunstrup, Nicklas HeineHeller, GerwinStavrianidi, AndreyHellewell, SarahStecker, Mark M.Hellstrom, Karl ErikSteel, Laura F.Hellweg, Christine E.Stefanidou, Maria E.Hellwig, MichaelStefanini, LuciaHenderson, W. MatthewStefano, CaldaralliHennequin, ClaireStefano, CassanelliHenry, Michael E.Steinmetz, Michel O.Heo, Soo-JinStenbeck, GudrunHepworth, ChrisStephan, HolgerHer, Lu-ShiunStępień, ŁukaszHeras, JosephStevanato, PiergiorgioHermann, MarcelaSteven, SebastianHermanns, HeikeStevenson, BradleyHernández-Hernández, A.Stewart, Cameron R.Hernández-Rodríguez, CésarStewart, JamesHerndon, DavidStewart, JasonHerr, Deron RaymondStewart, Lamonica V.Herranz, BeatrizSteyn, FrederikHerrero, BaudilioStillaert, FilipHerrero, María JesúsStilli, DonatellaHerrero-Beaumont, GabrielStillman, MartinHershfinkel, MichalStingeni, LucaHertz, Daniel L.Stiuso, PaolaHess, AngelaStivrins, NormundsHesson, LukeStobdan, TseringHettinga, KasperStobiecki, MaciejHewitt, Kevin CecilStocco, CarlosHickey, Michael J.Stocker, RetoHigashi, TomomiStokes, Karen Y.Higuchi, KazuhideStone, KariHii, Charles S. T.Stone, Trevor WilliamHildreth, BlakeStonehouse, NicolaHildt, EberhardStopeck, Alison T.Hileman, LenaStopka, PavelHill, Hellen Z.Stopka, TomášHillard, CeciliaŠtorchová, HelenaHimeno, HyoutaStorer, Nicholas P.Hinds Jr., Terry D.Stork, Christian J.Hininger-Favier, IsabelleStout, David A.Hinkelbein, JochenStowasser, MichaelHinojosa, SilviaStowell, Kathryn M.Hinz, SebastianSt-Pierre, YvesHirabayashi, JunStratmann, BerndHiraga, ToruStrauss, SarahHirakawa, HidehikoStrillacci, Maria GiuseppinaHirano, KatsuyaStrohbach, AnneHirano, TomokoStroka, Deborah M.Hirano, TomoyaStrong, Cristina De GuzmanHirasawa, NoriyasuStrudwick, XantheHirasawa, TakashiStrzemiecka, BeataHirbe, Angela C.Studzinski, George P.Hirsch, PierreStumpel, Connie T. R. M.Hitchins, MeganStunes, Astrid KamillaHitomi, HirofumiSu, JianguoHla, TimothySu, RuiHo, Chi-TangSu, TingHo, Jar-YiSuarez-Cunqueiro, M. M.Ho, MengfeiSuau, PedroHo, Roger Chun-ManSubramaniam, PrasadHo, Shin-LonSudhir, Putty-ReddyHo, Wen-FuSugahara, TakuyaHo, Wing ShingSugawara, AkiraHoang-Vu, CuongSugimoto, NaotoshiHochberg, IritSugimoto, SachikoHöckner, MartinaSugimoto, YukioHodgkinson, JamesSugimura, HaruhikoHoemann, CarolineSugita, NorikoHoff, RodneySugiura, DaisukeHoffmann, KlausSukhov, VladimirHoffmann-Benning, SusanneSukocheva, OlgaHofmann, JohannSun, FengjieHogstrand, ChristerSun, JieHöhfeld, JörgSun, LiangHojjat-Farsangi, MohammadSun, NaweiHolalu, SrinidhiSun, QianHolder, Alvin A.Sun, YingjieHolen, IngunnSun, YuanHolland, OliviaSun, YuxiangHöllig, AnkeSundar, Isaac KirubakaranHollomon, Mario GSundar, ReshmaHollywood, MarkSunseri, FrancescoHolmes, Dawn E.Suntharalingam, K.Holmøy, TrygveSurapathrudu, KanakalaHolst, OttoSurdacki, AndrzejHolt, JeffreySurguchov, AndreiHolt, Scott M.Sussman, Michael R.Holzmann, KlausSutaria, DhruvitkumarHoma, JoannaSutherland, John B.Homma, Miwako KatoSuzuki, HideoHon, Kam-LunSuzuki, Jon Y.Hondermarck, HubertSuzuki, NobuhiroHong, YonggeunSuzuki, TakehiroHood, SeanSuzuki, TakuyaHooijberg, ErikSvenningsen, Åsa FexHooper, CorneliaSvensson, Katrin J.Hopperton, KathrynSwamy, GaneshHori, YuichiroSwarup, VimalHori, YuuichiSweazea, KarenHorohov, David W.Sweetman, MartinHorrocks, A. RichardSwevers, LucHorvath, David P.Świeca, MichałHorvath, GyörgySwindle-Reilly, Katelyn E.Hosick, PeterSyed, NeloferHoskins, ClareSyed, ViqarHosmane, NarayanSykes, Erin K.Hosohata, KeikoSykora, PeterHosur, VishnuSylwester, SlusarczykHou, AnfuSytykiewicz, HubertHou, Chien-WeiSzablewski, LeszekHouen, GunnarSzabo, AronHoushmand, SinaSzarmach, ArkadiuszHouston, JessicaSzebeni, Gabor JanosHouston, Mark C.Széll, MártaHove-Jensen, BjarneSzewczyk, BernadetaHoving, HildeSzewczyk, KatarzynaHowarth, GordonSzilagyi, AndrasHowell, ViiveSzklarczyk, DamianHoying, James B.Szmidt, Alfred E.Hrabal, RichardSzopa, AgnieszkaHribal, MartaSzostak, Michael P.Hrobárik, PeterSzumny, AntoniHrstka, RomanSzyszkowicz, MieczyslawHsia, Shih-MinT. M. De Rosales, RafaelHsiao, GeorgeTababat-Khani, PoyaHsieh, Hsi-LungTabata, YasuhikoHsieh, James J.Tabrizi, Mojgan AghazadehHsieh, Tusty-JiuanTacconi, CarlottaHsieh, Yi-HsienTachibana, YuyaHsin, Kun-YiTadić, VanjaHsu, Erin L.Taghibiglou, ChangizHsu, Kuo-ShengTaguchi, AyumuHsu, Shu-haoTaguchi, Y. H.Hsu, ToddTahimic, CandiceHsu, Ya-LingTaiho, KambeHsueh, Yi-HuangTaiki, AoshiHu, ChaoTajima, ShojiHu, Michael Sung-MinTajima, SoichiroHu, MinTakabe, KazuakiHu, Ming-ChangTakac, TomasHu, Wei-GangTakagi, KiyoshiHu, YangTakahashi, DaisukeHuang, Chiu-jungTakahashi, HirokiHuang, Guan-JhongTakahashi, Ryou-uHuang, HaoTakahisa, KanekiyoHuang, Jason C.Takaki, AkinobuHuang, JianTakaki, ManabuHuang, LeiTakano, KatsuraHuang, Meng-YuanTakano, ToshiyukiHuang, Nai-KueiTakata, MinoruHuang, ShaobaiTakatsuji, HiroshiHuang, Shyh-ShyunTakatsuka, JunHUANG, Wen-ChinTakayama, KeiHuang, Wen-LiiTakayama, KenichiHuang, WenlinTakayama, YoshiharuHuang, XingfengTakegahara, NorikoHuang, Yan-JangTakeoka, ShinjiHuang, Ying-HsienTakumi, ShigeoHuang, Yu-TzuTallerico, RossanaHuber, SamuelTalò, GiuseppeHuber, VeronicaTalukder, Shyamal KrisnaHuettmann, FalkTamama, KenichiHughson, Michael D.Tamara, VarcoeHulot, Jean-SébastienTamás, LadislavHulse-Kemp, AmandaTamayol, AliHung, Jan-jongTamma, GraziaHunt, Piper ReidTan, Bee K.Huppert, VolkerTan, Dun-XianHuppi, KonradTan, Gene S.Huppler, Anna R.Tan, Sih MinHura, TomaszTan, XiangshiHusnjak, KoraljkaTanabe, ShihoriHutchinson, EdwardTanaka, AyumiHutmacher, DietmarTanaka, HiromitsuHüttenberger, DirkTanaka, HiroyukiHuwiler, AndreaTanaka, NobuyukiHwang, In KooTanaka, TakashiHwang, Ki-ChulTanaka, TakujiHwang, Yong-sicTanaka, TamotsuHwang, YongsungTanaka, TomoakiHymowitz, SarahTanaka, ToshiakiHyun, Kyung-YaeTang, Ming-JerHyun, Sang-HwanTang, YuIaccino, EnricoTanoi, KeitaroIacobellis, GianlucaTanonaka, KouichiIacono, PierluigiTan-Wilson, Anna L.Iadarola, PaoloTao, ShashaIbeanu, GordonTao-Hsin, TungIbiza, SalesTappia, Paramjit S.Ichikawa, HiroshiTarafder, SolaimanIchimura, KazuyaTarasova, Nadya I.Ieraci, AlessandroTarn, Woan-yuhIglesias-Fernández, RaquelTarr, D. EllenIjima, HiroyukiTarro, GiulioIjiri, DaichiTaruno, AkiyukiIkeda, MasahiroTashima, ToshihikoIkegaya, NaokiTashiro, HirotakaIles, RayTata, Ada MariaIlies, Marc A.Tate, Rothwelle J.Iliopoulos, KonstantinosTateishi, KensukeIlluminati, GiulioTatsuke, TsuneyukiIlmer, MatthiasTatullo, MarcoIm, Seung-SoonTaube, JoeIm, Sin-hyeogTaubert, AndreasImai, KazushiTaura, KojiroImaizumi, TakatoTausk, FranciscoImlach, Wendy L.Tavares, AnthonyInaba, HiroshiTavazzi, BarbaraInagaki, NoritoshiTawil, Bill J.Inamura, KentaroTaylor, CarolineIncitti, TaniaTaylor, ElaineIndiveri, CesareTaylor, GrahamIngels, HeleneTaylor, Juliet M.Ingley, EvanTaylor, KathrynIngram-Smith, CherylTaylor, MatthewIn-Jung, KimTaylor, T. MatthewInman, SharonTazawa, HiroshiInoue, AtsukoTe Riele, HeinInselman, AmyTe Velthuis, AartjanIntaglietta, MarcosTedbury, PhilipInui, HideyukiTedesco, SerenaInuzuka, HiroyukiTee, Andrew R.Ioanna, AndreadouTeeri, TeemuIordanskiy, SergeyTeets, NicholasIorio, EgidioTeige, MarkusIorio, MarilenaTeixeira, Renake N.Iorio, Ronald M.Teixidó, ElisabetIorizzo, MassimoTejedo, Juan RIqbal, M. AnwarTejero, JesúsIqbal, Tariq H.Tellone, EsterIraci, NunzioTelysheva, GalinaIrato, PaolaTempleton, Dennis J.Irimia, DanielTencerova, MichaelaIrimura, TatsuroTeng, Ru-JengIrvine, Katharine M.Teng, ShaoleiIsaji, ShujiTenta, RoxaneIsani, GloriaTentolouris, NicholasIsemura, MamoruTeodoro, Jose G.Isenberg, Jeffrey STeoh, EugeneIshibashi, KenichiTeotia, PoojaIshihara, HisamitsuTer Beek, JosyIshihara, KengoTerajima, MasanoriIshii, IsaoTerracciano, DanielaIshii, KazunariTerrier, OlivierIshii, TetsuroTerry, Randall G.Ishikawa, KiyotakeTeruel-Montoya, RaúlIshikawa, San-eTerzaghi, WilliamIshimoto, TakatsuguTeschke, RolfIshita, ChatterjeeTestai, Fernando D.Ishiyama, KatsuyaTeusch, NicoleIshizaki, TakumaTevosian, Sergei G.Islam, M. NurulTezil, TugsanIslam, Md SorifulThakkar, PrashantIsshiki, YoshiakiThakur, ChitraIta, KevinThannhauser, Theodore W.Ito, Ken-ichiTheile, DirkIto, KouseiTheiss, Arianne L.Ito, ShosukeThelin, EricItoh, Masanori T.Thi, Mia MiaItoh, YoshifumiThiel, GeraldItou, JunjiThiel, VolkerIttner, ArneThiem, KathrinIvancic Santek, MirelaThierry, HauetIvanov, Alexander VThijs, SofieIvanova, LyudmilaThilmony, RogerIvask, AngelaThiriet, MarcIwaizumi, Masakazu G.Thiruvengadam, MuthuIwamori, MasaoThomas, Seddon Y.Iwamoto, SadahikoThomas, T. JohnIzawa, TakashiThomas, WayneIzawa, TakeshiThompson, CristaIzquierdo-Useros, NuriaThompson, Loren P.Izsvák, ZsuzsannaThompson, Van PurdyIzumiya, YoshihiroThoms, Kai-MartinJablonska, EwaThorpe, ChavaunneJackson, ChristopherThounaojam, MenakaJackson, DanielThreadgill, MichaelJacob, FrancisThreapleton, Diane ErinJacobs, Aaron T.Thrivikraman, GreeshmaJacobs, EnnoThunström, ErikJacobs, MiriamTian, BinJacobson, LaurenTiemann-Boege, IreneJacova, ClaudiaTiffen, Jessamy C.Jacquet, AlainTiidus, PeterJadavji, Nafisa M.Tille, Jean-ChristopheJaffar, ZeinaTilley, MichaelJagiello, KarolinaTimme-Laragy, Alicia R.Jahandideh, SamadTimoshenko, Alexander V.Jain, GauravTimperio, Anna MariaJain, RachitTinuper, PaoloJaing, Tang HerTiso, NatasciaJaiswal, Amit K.Titorenko, Vladimir I.Jaiswal, Ashvin R.Tiwari, SanjayJaiswal, J. K.To, Kin-YingJakobi, TobiasTobimatsu, YukiJakovcevski, MiraTobita, HiroyukiJakubikova, JanaToden, ShusukeJakubzick, Claudia V.Todorovic, SlobodanJaligot, EstelleTogbe, DieudonnéeJames, EuanToh, Wei SeongJanakiraman, HarinarayananToldra, FidelJanda, TiborToledo-Ortiz, GabrielaJanecka, AnnaTolkach, YuriJang, An-SooTolosa, EzequielJang, SeonghoeTomar, DhanendraJanga, MadhusudhanaTomasetti, CarmineJantschitsch, ChristianTomaz, Cândida TeixeiraJanuchowski, RadosławTombesi, SergioJanusz, MariaTomita, HiroyukiJaradat, NidalTomitaka, AsahiJara-Palacios, M. JoséTommasini, Steven M.Jareño-Esteban, José JavierTomuta, IoanJarząb, BarbaraToniolo, Antonio Q.Jat, Parmjit S.Töreyin, HakanJaumot, JoaquimTorras, JoanJawien, JacekTorres Lagares, DanielJayant, Rahul DevTorres López, M^a^ IsabelJayaram, LataTorres Perales, CarolinaJazirehi, AliTorres-Aleman, IgnacioJellyman, J. K.Torres-Benito, LauraJeltsch, AlbertTorres-Reveron, AnnelynJembrek, Maja JazvinšćakTorzewski, MichaelJena, Bhanu P.Toth, EricJena, PrasantTouboul, CyrilJendelova, PavlaTous, NuriaJeng, Jiiang-HueiTovoli, FrancescoJenkins, Jill A.Toy, RandallJenkins, Johnie NortonToyoda, AtsushiJensen, BenjaminToyoda, YuJensen, Boye L.Trabace, LuigiaJensen, EricTramontano, DonatellaJensen, Lars HenrikTran, KimJensen, Randy L.Tran, PamelaJeong, DaewonTran, Thach D.Jeong, Keun-YeongTranbarger, TimothyJeong, Kyoung YongTrant, JohnJeong, Seon-YongTrapani, Joseph A.Jeschke, MarcTrautinger, FranzJeschke, UdoTravlos, IliasJesenak, MilosTraw, Milton BrianJesionowski, TeofilTrdan, StanislavJha, AmitTreesukosol, YadaJi, PengTreglia, GiorgioJiang, Shu-YeTrejo, JoannJiang, SizunTremblay, Marie-ÈveJimenez-Rondan, FelixTrenti, AnnalisaJin, Dong-HoonTrentini, AlessandroJin, FanTresguerres, JesúsJin, JiefuTrevisan, AndreaJin, RanTrevisan, GiustoJin, XinTrevisi, LuciaJinesh, Goodwin G.Triaca, VivianaJinnouchi, HideakiTrichet, ValérieJo, Young SukTricoire, LudovicJob, DominiqueTrif, MonicaJobling, PhilTrilling, MirkoJoers, ValerieTringali, CorradoJohansson, PegahTrivedi, Chinmay M.Johansson, StaffanTrivedi, DarshanJohn, RohanTrivedi, Evan R.John, VargheseTroczka, Bartlomiej J.Johnson, Thomas V.Troiano, GiuseppeJohnson, XenieTroise, Antonio DarioJohnston, MichaelTrojanowicz, BoguszJohnstone, ElizabethTrombetta, DomenicoJoles, JaapTroppmair, JakobJolly, PawanTrovato, GuglielmoJones, T. BuckyTrovik, JoneJonkers, IlseTrujillo, MarcoJonnalagadda, Subash C.Trujillo-Rodríguez, María JoséJonsson, Lisbeth M.V.Truman, AndrewJonušienė, VioletaTrummer, OliviaJoo, Kyeung MinTruong Phuoc, NghiaJordan, Stanley C.Tsai, JamesJordanova, Ekaterina S.Tsai, Kun-ChihJørgensen, Anders PalmstrømTsai, Kuo-WangJørgensen, Jens OttoTsai, Shih-JenJorrin-Novo, Jesus V.Tsang, Suk-YingJosé Hipólito, Isaza MartínezTschierlei, StefanieJoshi, BharatTse, William K. F.Joshi, Pushpa RajTseng, Ching-LiJouanneau, SulivanTsirigotis, PanagiotisJourdes, MichaelTsitsilonis, Ourania E.Jovanović, AleksandarTsoulfas, GeorgeJozic, IvanTsubaki, KazunoriJuan, OscarTsuchiya, YuichiJuarranz, ÁngelesTsuji, MasahiroJuhasz, BelaTsuji, ShojiJullian, NathalieTsunematsu, TomomiJulve, JosepTsurkan, Mikhail V.Jun, Hee-SookTu, MinJung, Chol-HeeTu, ThomasJung, KlausTuan, Tai-LanJung, Sung-ChulTuda, MidoriJunier, Marie-pierreTullio, VivianJurewicz, JoannaTunaru, SorinJurikova, TundeTuomisto, JoukoJust, NathalieTuorto, FrancescaJust, SteffenTurck, NatachaJuszczak, LesławTurco, JeniferJuszczyński, PrzemysławTuresky, RobertKabala-Dzik, AgataTuriel, MaurizioKaczor, Agnieszka A.Turnbull, D.Kadimisetty, KarteekTurpin-Nolan, SarahKadioglu, OnatTurrini, EleonoraKaeffer, BertrandTurroni, SilviaKageyama, YukioTuthill, Tobias J.Kagiya, TadayoshiTuttolomondo, AntoninoKahlert, ChristophTvrdik, PetrKahlert, UlfTyagi, RahulKai, HiroyukiTyakht, Alexander V.Kai, KentaroTyan, Yu-ChangKai, MihokoTyerman, Stephen D.Kaittanis, CharalambosTzakos, Andreas G.Kaji, ToshiyukiTzfadia, OrenKajiya, HiroshiUchida, ShizukaKakinuma, YoshihikoUchida, TakeshiKako, TetsuyaUchihashi, TakayukiKalani, AnuradhaUchio, YujiKale, AbhijitUeda, HiroshiKalinin, Vladimir I.Ueha, SatoshiKallunki, TuulaUeno, Shu-ichiKalogiannis, Konstantinos G.Ueno, TakafumiKalogirou, CharisUfnal, MarcinKamachi, KazunariUhal, Bruce D.Kamada, YoshihiroUhl, EberhardKamagata, KiyotoUlisse, SalvatoreKamal, Abu Hena M.Ulivieri, CristinaKamath, SandipUlloa, RitaKamato, DanielleUlrich, M.M.W.Kamiloglu, SenemUmberger, Reba A.Kamisuki, ShinjiUmehara, MikihisaKamitani, ShigekiUngefroren, HendrikKamiya, MitsunobuUnno, KeikoKamnev, AlexanderUpadhyaya, InduKamolz, LarsUrban, Jill P.G.Kamphuis, Lars G.Urbanelli, LorenaKanakkanthara, ArunUrbano, A. M.Kanamori, TakaoUsui, MichihikoKanaoka, MasahiroUusitupa, MattiKanasaki, KeizoUysal, UtkuKandimalla, RajuVáclav, BrázdaKanemori, MasaakiVáclavíková, RadkaKang, Chang MooVago, RiccardoKang, ChenVähä-Koskela, MarkusKang, HeeVaidya, BhuvaneshwarKang, HunseungVaidyanathan, GanesanKang, LeiValdes, AnaKang, SinyoungVale, NunoKang, Tae-HongValensin, DanielaKang, WeiValentina, VairaKanie, OsamuValentino, MarioKanik, MehmetValentová, KateřinaKaňková, KateřinaValentovic, MonicaKannan, RamValenzuela, RodrigoKano, MitsuyoshiValério, ElisabeteKant, MerijnValero, DanielKanvil, SadiaVali, PayamKanzaki, HiroyukiValles, Soraya L.Kao, Erl-ShyhValverde, FedericoKapitsinou, PinelopiVan Aken, OlivierKaplan, CraigVan Ballegooijen, Adriana JKappachery, SajeeshVan Bergen En Henegouwen, Paul M.P.Kappeler, LaurentVan Beuningen, Henk M.Kappen, ClaudiaVan Bever, YolandeKapus, AndrásVan Coppenolle, FabienKarakasidis, T. E.Van Cruchten, Steven J.Karakochuk, CrystalVan Cutsem, PierreKaraman, SinemVan De Ven, RienekeKararigas, GeorgiosVan Den Bergh, HubertKarlic, HeidrunVan Den Noort, M. W.M.L.Kårlund, AnnaVan Der Kuyl, AntoinetteKarmakar, MausitaVan Der Laarse, Willem J.Karni, RotemVan Der Mei, Ingrid A.f.Karpas, AbrahamVan Der Spoel, Aarnoud C.Karran, PeterVan Der Straaten, TaharKarras, SpirosVan Der Veer, Eric P.Karreth, Florian A.Van Der Weijden, RenataKarvinen, SiraVan Eps, Andrew Van EpsKaschina, ElenaVan Eys, Guillaume J.Kashfi, KhosrowVan Geijlswijk, Ingeborg M.Kashiwagi, ShinichiroVan Hall, ThorbladKashiwakura, IkuoVan Leeuwen, HansKashman, YoelVan Lent, Peter LEMKashofer, KarlVan Loo, Hanna M.Kasiotis, Konstantinos M.Van Noorden, CornelisKasoju, NareshVan Rijn, Richard M.Kassiri, ZamanehVan Roosbroeck, KatrienKasten-Jolly, JaneVan Scherpenzeel, MoniqueKataoka, NaoyukiVan Spaendonck-Zwarts, K. Y.Katarina, StroffekovaVan Vliet, SandraKatase, NaokiVan Waardenburg, R. C. A. M.Kathuria, HimanshuVancurova, IvanaKato, Takamitsu A.Vandenwijngaert, SaraKato, YasumasaVanderheyden, PatrickKatoh, MasaruVandier, ChristopheKatsantonis, DimitriosVandooren, JenniferKatsarava, RamazVanduffel, WimKatsila, TheodoraVangelista, LucaKatsoris, PanagiotisVankemmelbeke, MireilleKaufman, BrettVankova, RadomiraKaufmann, DorotheaVanneaux, ValérieKaunas, Roland R.Vanneste, SteffenKauppinen, AnuVapaatalo, HeikkiKaur, JasmineVaquero, JavierKawabata, TakeshiVarando, GherardoKawada, ManabuVarela-Nieto, IsabelKawahata, KimitoVasaikar, SuhasKawai, Vivian K.Vashist, Yogesh KumarKawai, YoshichikaVasicek, OndrejKawanami, DaijiVasin, Mikhail V.Kawano, KouichiroVassalle, CristinaKawano, TomonoriVassilaki, NikiKawasaki, HideyaVassilopoulos, GeorgeKawasaki, IchiroVaughan, Roger A.Kawasumi, MasaokiVaughn, ByronKawiak, AnnaVaughn, MathewKayama, MasazumiVaya, JacobKayisli, UmitVaz, Deisi AltmajerKazeto, YukinoriVaz, JosianaKazlauskas, AndriusVazquez-Manrique, RafaelKazunori, KadotaVecchione, CarmineKearsey, StephenVecoli, CeciliaKedrov, AlexejVeenstra, Richard D.Keku, TemitopeVeeramani, SureshKeller, SimonaVeiga-Lopez, AlmudenaKelley, DarshanVeilleux, AlainKelley, EricVeis, ArthurKelly, Clive AnthonyVejux, AnneKenna, Dervla T.D.Velasco Ortega, EugenioKenneth Matthew, ScaglioneVelasco, GuillermoKerwin, Rachel E.Veldkamp, ChristopherKeshteli, A. H.Veleeparambil, ManojKessler, SonjaVelena, A.Kevadiya, BhaveahVelivelli, SivaKevadiya, BhaveshVellante, FedericaKeyaerts, MarleenVelliyagounder, KabilanKhadka, ManojVemula, HarikaKhalid, SymaVenditti, AlessandroKhalil, AbdelouahedVento, RenzaKhaliulin, IgorVenuti, AldoKhallouki, FaridVerdaguer, DolorsKhan, MohsinVerdoucq, LionelKhan, MuhammadVergadi, EleniKhan, MushfiquddinVergara, DanieleKhandaker, MorshedVerges, MarcelKhare, VineetaVerhage, LeonieKhasawneh, FadiVerhoeven, AdrieKhatir, Dinah S.Vernetti, Lawrence A.Khatodia, SurenderVerron, EliseKhatri, KshitijVerstraete, AlainKhatri, MaheshVervaet, BenjaminKhetani, SalmanVeskoukis, Aristidis S.Khoo, Bee LuanVesper, StephenKhoobchandani, MenkaVetere, AmedeoKhoshmanesh, KhashayarVetter, Stefan W.Khotimchenko, Maxim Y.Vetvicka, VaclavKhurshid, ZohaibVetvickova, JanaKibe, ToshiroViapiana, AgnieszkaKibriya, MuhammedVicente, ClaudiaKichkailo, AnnaVicente, Joana G.Kido, Mizuho A.Vicente, LauraKiely, PatrickVictoni, TatianaKilari, SreenivasuluVictor, BrunoKillilea, DavidVideira, Romeu AntónioKim, Bong-SungVieira Ribeiro, João RuiKim, Dong JoonVieira, Margarida C.Kim, E. EdmundViejo-Borbolla, AbelKim, Haeng-HoonViel, SébastienKim, HangunViennois, EmilieKim, Hong-HeeViggiano, AndreaKim, HoonViggiano, DavideKim, Hun SikVigueira, CynthiaKim, Hyun UkVijayavenkataraman, S.Kim, Hyung-GooVilageliu, LLuisaKim, Hyung-JoonVilela, AliceKim, InKyeomVilla, Roberto FedericoKim, JaehanVilla-Bellosta, RicardoKim, JaehwanVillegas-Sepúlveda, NicolasKim, Jeong-RaeVincent, Jean-louisKim, Ji YeonVinciguerra, ManlioKim, Jin MoonVinnedge, Lisa PrivetteKim, Jin-KyungVinogradov, EvgueniiKim, JinuViola, IvanaKim, Jin-WookVirtue, SamKim, JoungmokVisani, MichelaKim, Jung-AeVisentin, MicheleKim, JwaJinVisioli, FrancescoKim, Ki HyunVisser, JacobKim, Kil-SooVisser, LydiaKim, Kyoung SooVita, RobertoKim, KyounghyunVitale, GiovanniKim, Kyung-suVítámvás, PavelKim, Nam-JungVitetta, LuisKim, Sang-WeVittorioso, PaolaKim, SunggilVivash, LucyKim, Sung-HoonVladimirov, VladimirKim, Sun-JuVlahou, AntoniaKim, Tae IlVliagoftis, HarissiosKim, Tae-AugVo, Minh D.Kim, Woo-YangVodicka, PavelKim, Young JoVoelkers, MirkoKim, Yun-baeVogel Ciernia, AnnieKimbrel, ErinVogelaar, Christina FranciscaKimler, BruceVogt, MartinKimura, MayumiVoitsekhovskaja, Olga V.Kimura, NobuyukiVolk, DavidKimura, TomokiVolk, Susan W.Kinaciyan, TamarVolz, David C.King, Chih-YenVon Knethen, AndreasKing, Irena B.Von Schacky, ClemensKinghorn, Kerri J.Von Wright, AtteKingsford, CarlVona, BarbaraKinsey, William H.Vonk, Lucienne A.Kirby, Lynn GuertinVorsters, AlexKirker, GrantVoss, MatthiasKirkham, M. B.Vossen, Jack H.Kirsch, ThorstenVostinaru, OliviuKiselev, K. V.Vrana, Nihal EnginKishigami, SatoshiVriend, JerryKishimoto, YoshimiVrsaljko, DomagojKita, HirohitoVrtala, SusanneKitaoka, SatoshiVu Bac, NamKitatani, KazuyukiVucenik, IvanaKitchen, PhilipVuckovic, DajanaKittaka, AtsushiVugrek, OliverKiyan, YuliaVulliamy, ThomasKlampfer, LidijaVuong, Thu V.Klampfl, StefanieVuorimaa-Laukkanen, ElinaKlaus, SusanneWada, JunKlein, David C.Wade, Charles E.Klein, StefanWadehra, MadhuriKlempt, MartinWadsak, WolfgangKleppe, LeneWagner, AnikaKlimaschewski, LarsWagner, DavidKline, KimberlyWagner, Gerd K.Kłoda, KarolinaWahlestedt, ClaesKlokov, Dmitry Y.Wahli, WalterKlontzas, MichaelWahyudi, HendraKlose, JohannesWaldmeier, FelixKlosterman, Steven J.Walentek, PeterKlug, JörgWaleron, KrzysztofKlutsch, JenniferWalker, Chandler L.Kluza, JéromeWalker, Douglas G.Kmetič, IvanaWalker, JessicaKmiec, EricWallace, DavidKmiec, Eric B.Wallace, NicholasKmiecik, JustynaWalne, Amanda J.Knaggs, RogerWalsh, LaurenceKnecht, HansWalters, DianneKnetsch, Menno L. W.Walts, Ann E.Knetsch, Menno L.W.Wan, LeiKnežević, Sanda VladimirWan, ShibiaoKnight, Jason S.Wan, XuehuaKniss, Douglas A.Wanders, DesireeKnölker, Hans-JoachimWang, Chao-MinKnott, K. EmilyWang, Chi ChiuKnutsen, SveinWang, Ching-ChiungKobayashi, HidekiWang, Chrong-ReenKobayashi, SatoruWang, ChuanFengKobayashi, YayoiWang, DandanKobayashi, YoshiroWang, DavidKobayashi, YutaroWang, DongKobeissy, Firas H.Wang, Feng-ShengKöbel, MartinWang, GuozhengKocarnik, Jonathan M.Wang, HaiKoch, AlexanderWang, HuaiminKoch, MarcoWang, J.Koduru, Srinivas VWang, James Q.KOENEN, Rory R.Wang, JenniferKoga, Jun-ichiroWang, Jian-WenKohanbash, GaryWang, JunKöhl, GudrunWang, JunjieKohl, MatthiasWang, LiangKohri, MichinariWang, MeinanKoivisto, AriWang, Peng-HuiKojima, MasamiWang, PiwenKojima, NaoyaWang, Raymond Y.Kojima, ShihokoWang, RichardKojima-Yuasa, AkikoWang, Robert Y. L.Kok, DieuwertjeWang, San-LangKok, Victor C.Wang, ShengKok, WouterWang, Sheng-FanKokabu, ShoichiroWang, Shyi-wuKolbe, L.Wang, Tian-LiKoller, MartinWang, Tong-HongKoloniuk, IgorWang, TuoKolpen, MetteWang, Tzu-haoKomasa, SatoshiWang, Wei-LienKomatsu, DavidWang, Wei-LungKomine, MayumiWang, WentianKomoike, YutaWang, XiaofeiKomorowski, JanWang, XiaohongKondo, EisakuWang, XiaowanKondo, HidehiroWang, XinKoneru, BhuvaneswariWang, XinkunKong, WeiliWang, XinwenKonig, HeikoWang, XipingKoning, Dirk DeWang, XuejunKonno, KatsuhiroWang, YanchangKonopka-Postupolska, D.Wang, YumengKonstantinov, Spiro M.Wang, YunmeiKontny, UdoWang, YunxiaoKontogiannatos, DimitriosWang, YuqiKooiman, KlazinaWang, ZhanxiangKootala, SujitWang, ZheKopecki, ZlatkoWang, ZhijieKopel, PavelWanibuchi, HidekiKoppe, Janna G.Ward, ChristopherKoppel, Barbara S.Ward, Peter AKorasick, David AWarnecke, AthanasiaKoren III, JohnWarren, SeanKornek, MiroslawWarzecha, ZygmuntKornprat, PeterWasternack, ClausKorrodi-Gregório, LuísWatanabe, AtsushiKorsching, EberhardWatanabe, EiichiKosaka, NobuyoshiWatanabe, MasatoshiKoskela, AliWaterman, CarrieKoskinen Holm, CeciliaWaters, Paula J.Koslicki, DavidWatkins, Adam J.Kosmala, ArkadiuszWatzka, MatthiasKosova, KlaraWaugh, Mark G.Kostakis, IoannisWawer, IwonaKostin, SawaWebb, Kimberly M.Kotake, ShigeruWeber, Michael J.Kotloski, RobertWeeden, Norman F.Kotsikorou, EvangeliaWege, StefanieKotta-Loizou, IolyWegner, Marthe-SusannaKotula-Balak, MalgorzataWei, ChangyongKoturbash, IgorWei, GanKounis, Nicholas GWei, QiuKoupenova, MilkaWei, RenKourist, RobertWei, Sai-nanKousoulas, Konstantin G.Wei, Shau-MingKoutelidakis, AntoniosWei, YufengKovac, StjepanaWeichert, JameyKovacic, HervéWeiergräber, MarcoKovács, Ákos T.Weigand, AnnikaKovalsky, IlonaWeigand, Julia E.Kovalskyy, DmytroWeihua, ZhangKovvasu, SuryaWeiser, Douglas C.Kowalska, MalgorzataWeivoda, MeganKowarik, Markus C.Welch, Babu GuaiKox, MatthijsWelch, CarrieKoyama, ShinWellberg, Elizabeth AKoyama, YuWeller, MichaelKozaki, AkikoWellner, UlrichKoziak, KatarzynaWelsch, RalfKozliak, Evguenii I.Welsh, MichaelKozlowski, Michael R.Wen, C.M.Kramer, EricWen, LuKramer, PhillipWen, XIAOMINKrammer, FlorianWende, WolfgangKrasnodembskaya, Anna D.Wendell, Stacy GelhausKratimenos, PanagiotisWeng, ChingfengKraus, VirginiaWeng, Mao-LunKrause, RolfdieterWeng, XiaoyuKrauss, Daniel J.Werstuck, Geoff H.Kregel, StevenWertz, Philip W.Křen, VladimírWest, James D.Krenek, PavelWest, TimKrepler, ClemensWestenfelder, ChristofKrisch, JuditWestin, GunnarKrischik, Vera AWhelan, Rebecca J.Krishna, SmritiWhirledge, ShannonKrishnan, LaxminarayananWhite, PeterKristensen, MieWhite, Stormi PulverKristian, TiborWhiteside, Teresa L.Krogan, Naden T.Whyard, SteveKrohn, SandraWickenden, Alan D.Krol, EwelinaWiczkowski, WieslawKroncke, Brett M.Widdison, Wayne C.Kropp, MartinaWidgerow, AlanKropski, Jonathan A.Wie, Myung-BokKrützfeldt, JanWiechec, EmiliaKruyt, Frank A. E.Wiechmann, AllanKu, Kang MoWiedenmann, JoergKu, SeockmoWiedman, GregoryKu, Seung-YupWiemer, Erik A.C.Kuan, Yu-HsiangWierenga, Corette J.Kuang, HuihuiWierzchowski, MarcinKubatka, PeterWieser, RotraudKubik, LukaszWiesner-Reinhold, MelanieKubiński, KonradWietrzyk, JoannaKubista, HelmutWigle, Jeffrey T.Kubo, TakanoriWild, EdwardKubo, TomohikoWildemann, BrittKubo, ToshioWilhelm, StefanKucukkal, TugbaWilkinson, Mark D.Kuhlmann, StellaWilkinson, RobertKui, BalázsWillcox, MarkKukula-Koch, WirginiaWilliams, AdinaKumar, BinodWilliams, Andrew R.Kumar, DhirendraWilliams, CeciliaKumar, GauravWilliams, DavidKumar, GokhleshWilliams, JohnKumar, ManishWilliams, Noelle S.Kumar, ManuWilliams, Thomas L.Kumar, MukeshWilliams, TrevorKumar, RajiveWilliamson, Sean R.Kumar, SathishWilmink, Johanna W.Kumar, SushilWilson, DouglasKumar-Singh, SamirWin, Khin YinKumazawa, YoshinoriWinkler, JohannesKumi-Diaka, JamesWin-Shwe, Tin-TinKumita, JanetWińska, KatarzynaKummer, WolfgangWinter, HarlandKundu, AishwaryaWinter, StephaneKunej, TanjaWinterbourn, ChristineKunjachan, SijumonWinters, BryonyKunkalla, KranthiWirbisky, SaraKunz, WolframWise, JohnKunze, GotthardWise, PetraKünzler, MarkusWis̈niowska, BarbaraKuo, Cheng-ChinWitkowski, MarcoKuo, Ping-ChungWitorsch, Raphael JayKuo, Po-LinWitowski, Jan SylwesterKuo, Tzong-FuWitten, Paul EckhardKurakula, KondababuWitt-Enderby, PaulaKurdowska, Anna K.Wittstock, UteKurenda, AndrzejWittwer, CarlKurokawa, MineoWnuk, MaciejKurschus, FlorianWobbe, LutzKurz, TinoWohleb, Eric S.Kuter, KatarzynaWohlert, JakobKuwabara, MasanariWojcieszyńska, DanutaKuyucu, SemanurWójkowska, D. W.Kuźnicki, JacekWojtaszek, PrzemyslawKveberg, LiseWojtkiewicz, JoannaKwa, Faith A.A.Wölfle, UteKwak, MinjungWolley, MartinKwakowsky, AndreaWong, Albert H.C.Kwan, Hiu-yeeWong, Boon-SengKwan, JamesWong, Ching-OnKweon, Oh-KyeongWong, Chung F.Kwiecien, Jacek M.Wong, ConnieKwon, HyokjoonWong, Pamela T.Kwon, Tae-HwanWong, Ronald J.Kwon, Tae-yubWong, ShunKwong, JosephWong, Vincent Kam WaiKyle, StuartWoo, So-YounLa Rocca, Renato V.Woodman, TimLa Russa, DanieleWoods, NicholasLaboisse, Christian L.Woolbright, BenLabruna, GiuseppeWorch, RemigiuszLachman, JaromirWorkman, VictoriaLacis, GunarsWozniak-Knopp, GordanaLadavière, CatherineWozniakowski, GrzegorzLærke, HelleWren, Brendan W.Lafontaine, DenisWright, CatherineLaforenza, UmbertoWright, ColinLagace, Thomas A.Wrzaczek, MichaelLagravère, Manuel O.Wu, ChengbiaoLagrimini, MarkWu, Chi-ReiLahiri, AmitWu, ChristinaLahiri, AmitabhaWu, HannahLahooti, HooshangWu, JiahuiLai, Julian C. L.Wu, Kevin C. W.Lai, Wen-Fu ThomasWu, Tzong-YuanLai, YandongWu, Wen-ShengLaird-Offringa, Ite A.Wu, YunqiLakhkar, AnandWullaert, AndyLakin-Thomas, Patricia L.Wydau, SandraLalonde, FrancoisWysocki, AnnetteLalowski, MaciejXavier, Cristina Pinto RibeiroLam, Hon-MingXi, LinLam, Wai-ChingXi, WeixianLAM, YulinXia, TianLamarche-Vane, NathalieXian, WaLamas, José RamónXiang, YuLamb, LauraXiao, JunhuaLamina, ClaudiaXiao, LiLaMontagne, MichaelXiao, YangyanLan, LanXiao, YuguoLandete, José MariaXiaoli, AlusLandreh, MichaelXie, HongLandreville, SolangeXie, JinghangLandry, Samuel J.Xie, QiLaner-Plamberger, SandraXie, Zhong-RuLange, DirkXin, DongyueLange, SigrunXin, JunnaLangkjær, NielsXin, RanLanz, Tobias V.Xing, JinchuanLaPres, John J.Xing, ZhuoLapucci, CristinaXiong, YeLardone, Patricia J.Xiong, ZhaohuiLarena, InmaculadaXu, DeyangLarijani, ManiXu, HainengLaRoque, JanXu, LeiLarraga, VicenteXu, PengfeiLarsen, Anders ChristianXu, QinqinLasky-Su, Jessica AnnXu, WangLassmann, HansXu, YanyiLassot, IrénaXu, YiLatella, GiovanniXu, YongjieLatorre, Juan DavidXuan, Tran DangLattanzio, RossanoYaegashi, HajimeLau, Wei LingYager, EricLaubach, Victor E.Yagüe, ErnestoLau-Cam, Cesar A.Yakovlev, Igor A.Launay, Jean MarieYamada, SohsukeLauretani, FulvioYamada, YasumasaLauro, ClotildeYamaguchi, MasayaLauschke, VolkerYamaguchi, SeijiLavandera, IvánYamaguchi, YubeLavarino, CinziaYamakoshi, KimiLavelli, VeraYamamoto, HideyukiLaviano, AlessandroYamamoto, YusukeLavoie, Jean-claudeYamamotova, AnnaLavrik, OlgaYamamura, SoichiroLawlor, KateYamanishi, KiyofumiLayh-Schmitt, GerlindeYamasu, KyoLazado, Carlo C.Yamauchi, YoheiLazarević, VladimirYan, BowenLe Bideau, FranckYan, DayunLe Bihanic, FloraneYan, DongqingLe Hir, HervéYan, QingLe Hir, RozennYanase, EmikoLe Ouay, BenjaminYang, Chin-YingLe, MinhYang, GuangLeanza, LuigiYang, HainingLebar, MatthewYang, Jae WookLebaron, RichardYang, Jen-ChangLebaron, SimonYang, Jenq-LinLebeck, JanneYang, JunLebedev, Albert T.Yang, Kuang-YaoLeborgne-Castel, NathalieYang, Rong-SenLebrun, BrunoYang, ShengyongLechel, AndreYang, Sherry X.Lechpammer, MirnaYang, TaoLeclercq, GuyYang, TianxinLedo, AnaYang, TuoLee, Byung C.Yang, Wei-hsiungLee, ChangWooYang, XinmaiLee, Che-HsinYang, YeLee, Chen-YuYang, YongkangLee, Choon-HwanYang, YunzeLee, Chul-HoYano, ShozoLee, Dong HunYano, TohruLee, Hak JongYao, JianLee, Heung-ManYao, JianboLee, HsinyuYao, JiayiLee, Huei-JaneYao, YanhuaLee, Je MinYar, MuhammadLee, J-HYasuda, KazukiLee, Jin-KyunYasuda, TakakoLee, Jong HunYasuike, MotoshigeLee, Joo HyoungYates, Nathanael J.Lee, Ju HeeYe, HaobinLee, Jun SikYe, ZhouLee, JungilYeckel, Catherine WeikartLee, JungwooYeh, Jwu-LaiLee, Kin Wah TerenceYeh, Sung-LingLee, Kyung-YilYeh, Yin-TingLee, KyuwanYektaei-Karin, ElhamLee, Maw-RongYeligar, SamanthaLee, Min-GeolYellen, GaryLee, Myung KooYen, Meng-ChiLee, Sang KilYeo, SynLee, Sang-HanYeung, Sai-Ching JimLee, SanghyeobYi, HaoweiLee, SangkwanYih, Ling-hueiLee, SoohongYilmazer, AçelyaLee, Su-JaeYin, JingwenLee, SukmookYin, KingsleyLee, Tzong-ShyuanYin, ShiLee, Wing-KeeYli-Mattila, TapaniLee, Yi-JangYochum, Gregory S.Lee, Yoon KwangYohei, ShirakamiLees, Watson J.Yokohira, MasanaoLefebvre, HervéYokoi, HidekiLeffler, JonatanYokoi, TsuyoshiLehmann, ChristianYokota, ShinsoLei, LeiYoneda, ToshiyukiLeibundgut-Landmann, S.Yonekura, ShinichiLeidenheimer, NancyYoneshiro, TakeshiLeiherer, AndreasYong, JeongsikLeitão, J. H. G.Yoo, Tag KeunLeitz, JeremyYool, AndreaLekli, IstvanYoon, Je-HyunLemu, Hirpa G.Yoong, MichaelLenaerts, KaatjeYoshida, HiroshiLenardon, Megan D.Yoshida, NaokoLendrem, DennisYoshida, YamatoLeng, RogerYoshimoto, KojiLennox, KimYoshino, JunLensink, Marc F.Yoshitomi, HiroyukiLeon, CarlosYou, SeungkwonLeon, JosefaYou, SylvaineLeonard, StephenYou, XiaonaLeone, AlessandroYoung, DavidLeoni, AlbertoYoung, G. BryanLeon-Reyes, AntonioYoung, Jason C.Leporatti, StefanoYoung, MarianLerm, MariaYovich, JohnLeroux, CarolineYu, AimingLeroy, BaptisteYu, Cheng-PingLeroy, JérômeYu, Hak KiLescrinier, EvelineYu, MeifangLeso, VerusckaYu, Yan PingLetizia, TrovatoYu, YangLeung, Chung-HangYu, YanlinLeung, Ricky Yuet-KinYu, YingjieLeustek, ThomasYu, Yong-mingLevesque, CelineYuan, ChunyanLevi, MosheYuan, HuipinLevinson, Ralph D.Yuan, XueLevraud, Jean-pierreYuan, ZhaoLevy, StevenYuan, ZhihongLewandoski, MarkYudoh, KazuoLewinska, AnnaYue, PatrickLi, AitaoYue, XiaojingLi, BenyiYue, XuyiLi, CalvinYuen, John WmLi, ChinYuh, Chiou-HwaLi, ChuanYukl, Erik T.Li, DapengYung, Hong-WaLi, DeyingYung, YuvalLi, FengYuzawa, YukioLi, GuohuiZabad, Rana K.Li, Hua-BinZąbek, AdamLi, HuiZabetakis, IoannisLi, HuinanZafar, MuhammadLi, JinpingZagidullin, NaufalLi, Kuo-BinZaid, HilalLi, LinZajonc, Dirk M.Li, MiaomiaoZakharian, EleonoraLi, MingŽáková, LenkaLi, NingjunZakrzewicz, DariuszLi, QiangZaky, AhmedLi, RobertZalupski, Mark M.Li, ShiwengZamberletti, EricaLi, Wen-WuZambrano-Wilson, Zambrano-WilsonLi, YanŻamojć, KrzysztofLi, YangZamyatnin, Andrey A.Li, YangmingZang, LiqingLi, YanjieZannetti, AntonellaLi, YimengZara, SeverinoLi, YujingZaric, Svetislav S.Li, ZheZarogoulidis, PaulLiagre, BertrandZarrelli, ArmandoLiao, Francesca-FangZarros, ApostolosLiao, Jen-ChungZarubaev, VladimirLiao, Jyh-feiZaza, GianluigiLiao, YichuZehbe, IngeborgLiao, Yi-JenZeilinger, CarstenLiapi, CharisZeilinger, KatrinLibik-Konieczny, MartaZeleny, ReinhardLicciardello, GraziaZeller, SebastianLidon, FernandoZellner, JohannesLifschitz, CarlosZeman, Daniel MeierLigon, Lee A.Zemel, MichaelLigterink, WilcoZenclussen, AnaLim, Jae HyangZendo, TakeshiLim, JunxianZervakis, Georgios ILim, KyuZeugolis, DimitiosLim, Lee WeiZevin, Alexander S.Lim, MingZhang, BoLim, Shiang Y.Zhang, CaiguoLim, SoyeonZhang, ChiLim, Sung-JigZhang, DuoLima, Sofia A. CostaZhang, HengyouLin, ChenZhang, LeiLin, Chih-ChienZhang, MengliangLin, Chih-LiZhang, QiuyangLin, DanielZhang, SuiLin, Hai-shuZhang, TaoLin, HoZhang, TuoLin, Jung-ChunZhang, WeiLin, KuiZhang, WeijieLin, Kwang-HueiZhang, WenhengLin, Li-HueiZhang, WenjunLin, NancyZhang, WenyuLin, Pei-HuiZhang, XuanLin, QianqianZhang, YaqingLin, Shankung LinZhang, YixiangLin, ShengdaZhang, YixuanLin, Ying-HungZhang, YumiaoLindemann, PeterZhang, YuningLinder, StigZhang, YunkaiLindner, RobertZhang, ZhiyongLing, HuiZhao, BaoyinLing, MauriceZhao, LinpingLingappan, KrithikaZhao, MeixiaLinhardt, RobertZhao, NingningLinninger, Andreas A.Zhao, QianLinseman, DanielZhao, QingnanLionetti, VincenzoZhao, XiaoaiLiou, Ying-mingZhao, XuebingLips, Katrin S.Zhbannikov, Ilya Y.Littlefield, Bruce A.Zheng, ShilongLitwin, MieczysławZheng, WenguangLiu, BaomingZheng, YueLiu, BotaoZhonping, XuLiu, Chao-LinZhou, BangjunLiu, Cheuk LunZhou, BenLiu, Chia-ChiZhou, Guo-PingLiu, ChunmingZhou, Heshan SamLiu, DelongZhou, JingjiangLiu, JiZhou, LinliLiu, JianfengZhou, QingxiangLiu, JinghuaZhou, WenchaoLiu, JunZhou, Xue-RongLiu, Jun-JenZhu, Guo-ZhangLiu, JunliZhu, JianhuaLiu, LiZhu, JinshengLiu, Pei-Yangzhu, LiandongLiu, RengyunZhu, QianhaoLiu, Shing-HwaZhu, Qiuyu MartinLiu, XiangshengZhu, WeiLiu, YangZhu, XiangweiLiu, YongqingZhu, YuwenLiu, YuZhukova, NataliaLiu, ZhenfengZhuo, Jia L.Liu, ZhixiaŽiarovská, JanaLiu, ZiqingZichel, RanLiWang, PatriciaZięba, AndrzejLjubimov, Alexander V.Zienius, DainiusLlobet-Navas, DavidZilioli, SamueleLlorens, EugenioZimmer, Warren E.Lloret, AnaZimmerlin, LudovicLo Muzio, LorenzoZiv, MeiraLo, Woo KuenZmijewski, MichalLoboda, AgnieszkaŻołnowska, BeataLobysheva, IrinaZonaro, EmanueleLöffler, MarkusZorc, BrankaLoganzo, FrankZorzano, AntonioLognay, GeorgesZosky, GraemeLoh, ZhixuanZoupa, MariaLohning, AnnaZucca, PaoloLoinard, CélineZuhl, MicahLombó, FelipeZukiewicz-Sobczak, W. A.Lonardo, AmedeoZüllig, RichardLongo, Dario LivioZuluaga, PaolaLongoni, GianlucaZuo, LiLönn, PeterZupko, IstvanLontok, ErikZuppinger, ChristianLoor, Juan JZutz, ChristophLooyenga, Brendan D.Zuvela, PetarLopes, FedericaZygadlo, Julio

